# Efficient precise knockin with a double cut HDR donor after CRISPR/Cas9-mediated double-stranded DNA cleavage

**DOI:** 10.1186/s13059-017-1164-8

**Published:** 2017-02-20

**Authors:** Jian-Ping Zhang, Xiao-Lan Li, Guo-Hua Li, Wanqiu Chen, Cameron Arakaki, Gary D. Botimer, David Baylink, Lu Zhang, Wei Wen, Ya-Wen Fu, Jing Xu, Noah Chun, Weiping Yuan, Tao Cheng, Xiao-Bing Zhang

**Affiliations:** 1 State Key Laboratory of Experimental Hematology, Tianjin, China; 2 Institute of Hematology and Blood Disease Hospital, Chinese Academy of Medical Sciences and Peking Union Medical College, Tianjin, China; 3 Center for Stem Cell Medicine, Chinese Academy of Medical Sciences, Tianjin, China; 4 Department of Stem Cell & Regenerative Medicine, Peking Union Medical College, Tianjin, China; 5 Collaborative Innovation Center for Cancer Medicine, Tianjin, China; 6 Tianjin Key Laboratory of Blood Cell Therapy and Technology, Tianjin, China; 7 Division of Regenerative Medicine MC1528B, Department of Medicine, Loma Linda University, 11234 Anderson Street, Loma Linda, CA 92354 USA; 8 Department of Orthopaedic Surgery, Loma Linda University, Loma Linda, CA USA

**Keywords:** CRISPR, Genome editing, Knockin, Homology-directed repair (HDR), Non-homologous end joining (NHEJ), Donor design, 293 T, Human induced pluripotent stem cells

## Abstract

**Background:**

Precise genome editing via homology-directed repair (HDR) after double-stranded DNA (dsDNA) cleavage facilitates functional genomic research and holds promise for gene therapy. However, HDR efficiency remains low in some cell types, including some of great research and clinical interest, such as human induced pluripotent stem cells (iPSCs).

**Results:**

Here, we show that a double cut HDR donor, which is flanked by single guide RNA (sgRNA)-PAM sequences and is released after CRISPR/Cas9 cleavage, increases HDR efficiency by twofold to fivefold relative to circular plasmid donors at one genomic locus in 293 T cells and two distinct genomic loci in iPSCs. We find that a 600 bp homology in both arms leads to high-level genome knockin, with 97–100% of the donor insertion events being mediated by HDR. The combined use of CCND1, a cyclin that functions in G1/S transition, and nocodazole, a G2/M phase synchronizer, doubles HDR efficiency to up to 30% in iPSCs.

**Conclusions:**

Taken together, these findings provide guidance for the design of HDR donor vectors and the selection of HDR-enhancing factors for applications in genome research and precision medicine.

**Electronic supplementary material:**

The online version of this article (doi:10.1186/s13059-017-1164-8) contains supplementary material, which is available to authorized users.

## Background

The ability to precisely edit genomes endows scientists with a powerful tool to interrogate the functionalities of any pieces of DNA in the genome of any species and it may also lead to the development of new therapies that can potentially cure numerous genetic diseases [1–3]. However, precise gene editing by homologous recombination is very inefficient, unless a DNA double-stranded break (DSB) is created at the targeting site, which increases homology-directed repair (HDR) mediated gene editing efficiency by ~1000-fold [4–6]. To induce DSB at a desired site, several technologies have been developed over the past decade, including zinc-finger nucleases (ZFN), transcription activator-like effector nucleases (TALEN), and the clustered regularly interspaced short palindromic repeats (CRISPR)/CRISPR-associated protein-9 nuclease (Cas9) system [1]. The CRISPR-Cas9 system has caught widespread attention due to its robust performance, simple vector construction, and multiplexability in manipulating genes [7, 8].

CRISPR is an adoptive immune system evolved in bacteria and archaea to fight against invading agents such as bacteriophages or plasmids [9]. Diverse CRISPR systems have been adapted for use in editing mammalian genomes [10–12]. Currently the most commonly used system is derived from Streptococcus pyogenes (Sp), which consists of a Cas9 endonuclease and two separate small RNAs, called tracrRNA and crRNA [13], that can be combined with a tetraloop to form a single guide RNA (sgRNA) [14]. SpCas9, which will be referred to henceforth as Cas9 for simplicity, cuts double strands of DNA to generate blunt-ended double strand breaks (DSBs) at 3 bp upstream of the NGG PAM (protospacer adjacent motif) under the guidance of sgRNA, which specifically recognizes the chromosomal loci of interest with 17–20 nucleotides (nt) [15, 16]. Cells repair DSBs primarily by two mechanisms: non-homologous end joining (NHEJ) and homology-directed repair (HDR). In comparison to NHEJ, which generates a knockout phenotype by introducing variable insertions or deletions (indels) at the DSB, the HDR pathway creates precise deletions, base substitution, or insertion of coding sequences of interest in the presence of a recombination donor flanked with right and left homology arms (HA). Thus, the HDR pathway can be exploited to facilitate correction of diseased genes, insertion of epitope tags or fluorescent reporters, and overexpression of genes of interest in a site-specific manner.

Using rationally designed sgRNAs, high-level gene knockout can be achieved in different types of cells [17–19]. However, improving the efficiency of precise CRISPR/Cas9-mediated gene editing or HDR-mediated knockin (KI) remains a major challenge, especially in human induced pluripotent stem cells and primary stem cells (iPSCs) [7, 20, 21]. Significant effort has been devoted to increasing knockin efficiency by improving targeting strategies, especially for insertion of a large DNA fragment. Previous reports used ZFN, TALEN, or CRISPR-Cas9 technology to knock in long DNA fragments via a homology-independent manner [22–26]. In these methods, the donor plasmid contains an endonuclease cleavage site and can be linearized in vivo when co-transfected with a specific endonuclease [22–26]. While these approaches are generic, they often lead to the integration of the entire donor plasmid and may induce mutagenic junctions caused by erroneous NHEJ, limiting the application potentials.

Hisano et al. [27] modified the donor plasmid by using short homologous sequences (20–40 bp) flanked by two sgRNA target sequences (also known as double cut donors), and observed efficient and precise integration of exogenous DNA into the predicted target locus in zebrafish. Similarly, donor vectors harboring microhomologous DNA ends have been used to edit human cells. However, the HDR efficiency before drug selection was not reported, which is likely to be less than 1% [27–29]. Longer HA is necessary for efficient HDR in human cells. It has been reported that HDR of oligonucleotides is most efficient when a single-stranded oligodeoxynucleotide (ssODN) template with 90 nt HA is used [30]. For HDR knockin of a large fragment, HA of ~0.2–0.8 kb have been reported to be necessary for transgene insertions and HA of up to 2 kb exhibited the optimal gene targeting efficiency in human iPSCs when a conventional circular donor is used [20].

However, no comprehensive studies have been reported on the comparison of the conventional circular plasmids and double cut donors in HDR efficiency and on the shortest HA that is required for high-level precise genome editing. Here we attempted to address these unanswered questions in 293 T cells and iPSCs. We further tested whether small molecules and other factors can increase HDR efficiency. We found that 20–30% HDR-mediated knockin can be achieved in human iPSCs using double cut donors with HA of 300–600 bp in length together with cell cycle regulators Nocodazole and CCND1 (also known as cyclin D1).

## Results

### A double cut HDR donor increases HDR efficiency in 293 T cells

**Fig. 1:**
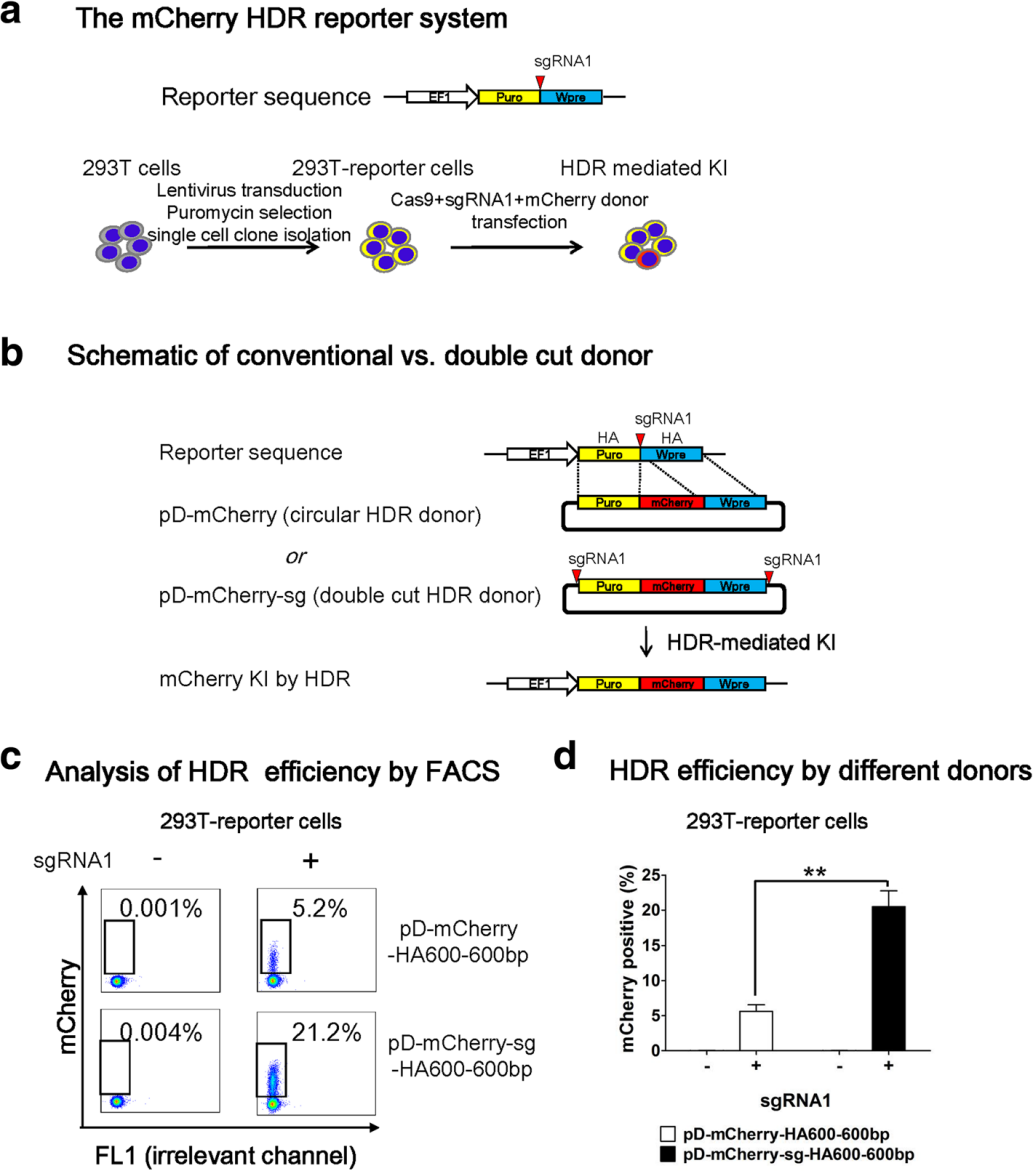
A double cut HDR donor considerably increases HDR efficiency in 293 T cells after CRISPR-mediated DSB. **a** *Schematic outline* of the mCherry HDR reporter system. A lentiviral vector Lenti-EF1-Puro-sgRNA1-Wpre was used to generate reporter cell line. The *red triangle* indicates a sgRNA1-PAM sequence that will guide Cas9 to create DSB. 293 T cells were transduced with the lentiviral vector at a low MOI. After transduction, cells were treated with puromycin (2 ug/mL) and single-cell cloning was conducted to generate reporter cell lines with Puro-sgRNA1-Wpre target sequence (293 T reporter cells). EF1 is the promoter that drives the expression of a puromycin resistance gene. Wpre is the woodchuck hepatitis virus posttranscriptional regulatory element. After co-transfection with promoterless mCherry donor and two plasmids encoding Cas9 and sgRNA1, the 293 T reporter cells use the donor to repair DSB by HDR pathway leading to the integration and expression of mCherry. **b** Design of promoterless mCherry HDR donors. pD-mCherry is a conventional circular HDR donor and pD-mCherry-sg is a double cut HDR donor in which the Puro-mCherry-Wpre cassette is flanked by two sgRNA1 recognition sequences. Puro (663 bp) and Wpre (592 bp) serve as left and right HA, respectively. To simplify naming scheme, the length of Puro and Wpre are unified as 600 bp and the tag HA600-600 bp indicates their HA length. **c** FACS analysis of 293 T reporter cells one week after co-transfection of Cas9 and conventional vs. double cut pD-mCherry donors, with or without sgRNA1. The portions of mCherry^+^ cells represent the HDR-mediated knockin efficiencies. **d** HDR efficiency by two different donors. n = 3; *error bars* represent S.E.M. Significance was calculated using the Student’s paired t-test: **P ≤ 0.01

First, we used the most commonly used 293 T cells to compare the two donor plasmid designs and examine the effects of homology arm (HA) length on HDR efficiency. To this purpose, we established a reporter system in 293 T cells (Fig. 1a). First, we lentivirally transduced 293 T cells at a low multiplicity of infection (MOI of 0.1–0.2) with Lenti-EF1-Puro-sgRNA1-Wpre, which contains a sgRNA1 recognition sequence between Puro and Wpre element. After puromycin selection, we conducted single-cell cloning and generated three 293 T reporter lines. Following co-transfection with a promoterless mCherry donor plasmid and two plasmids encoding Cas9 and sgRNA1, mCherry is knocked into the target locus by HDR and the cells become mCherry^+^ (Fig. 1b). Although NHEJ insertion of donor may occur in this system, these cells would remain mCherry^–^. As such, the portion of mCherry^+^ cells detected by flow cytometry (FACS) reflects HDR efficiency. We will address NHEJ integration in later sessions.

For correcting single nucleotide polymorphisms (SNPs) or generating precise point mutations, single-stranded oligodeoxynucleotides (ssODNs) have been successfully used as donor templates after creating a DSB [30–32]. For editing large pieces, double-stranded DNAs with relatively long HA are required to achieve relatively high efficiency [20]. The conventional donor is a circular plasmid carrying an insert flanked by two HA. Recently, polymerase chain reaction (PCR) products containing HA have also been used as donors due to its simplicity and lack of need for vector cloning [33, 34]. However, PCR products were similar to conventional donor plasmids in HDR efficiency, limiting its application potential (data not shown).

We hypothesize that in vivo cleavable donor plasmid can increase HDR; this can be achieved by sandwiching the donor vector with two sgRNA recognition sequences. When the Cas9/sgRNA complex surveys the genome and plasmids, it creates genomic DSB and linearizes donor plasmids simultaneously, thus synchronizing the demand and supply of homologous sequences and thereby increasing HDR. To test this idea, we compared the HDR efficiency using two types of donors: pD-mCherry, a conventional circular HDR donor and pD-mCherry-**sg**, a double cut HDR donor in which the Puro-mCherry-Wpre cassette is flanked by two sgRNA1 recognition sequences (Fig. 1b). In this manuscript, we tagged **sg** in the donor plasmid name to distinguish it from the commonly circular donor. In the two template plasmids, Puro (663 bp) and Wpre (592 bp) are identical and serve as left and right HA, respectively. To simplify the naming scheme, we unified the length of Puro and Wpre as 600 bp and tagged a label of HA600-600 bp to the two donors to indicate their HA length. As expected, we observed a fourfold increase in the portion of mCherry^+^ cells with double cut donor pD-mCherry-sg-HA600-600 bp compared to pD-mCherry- HA600-600 bp (Fig. 1c, d).

### High HDR efficiency is achieved in 293 T cells by double cut HDR donors even with short homology arm

**Fig. 2:**
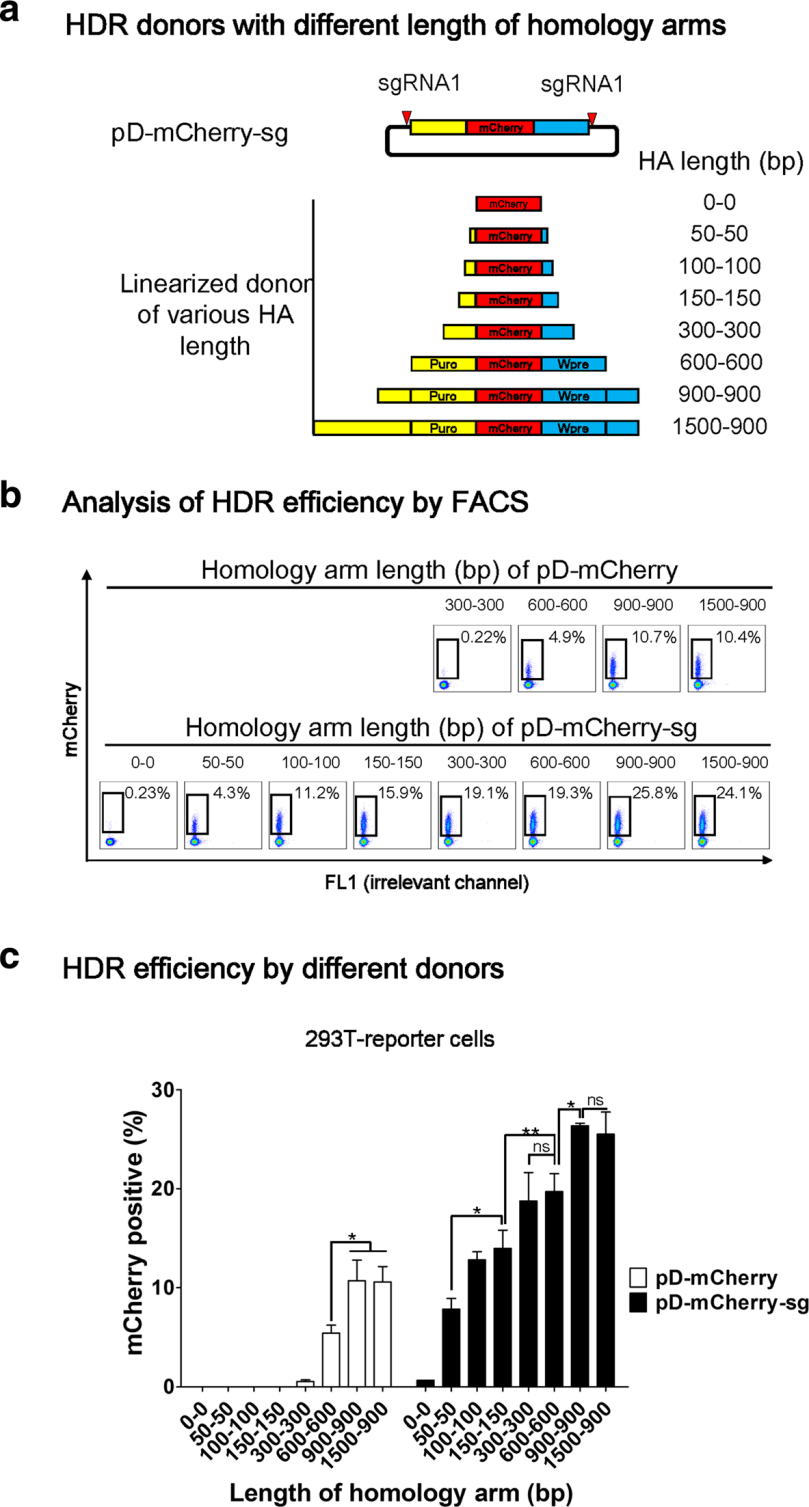
High HDR efficiency is achieved in 293 T cells by double cut HDR donor even with short HA. **a** *Schematic outline* of pD-mCherry-**sg** (double cut HDR donor) with HA in the range of 0–1500 bp in length. The *red triangle* indicates a sgRNA target sequence. The left arm is marked as *yellow* and the right arm as *blue*. **b** Determination of the HDR efficiency by FACS. 293 T-reporter cells were analyzed one week after co-transfection of Cas9, sgRNA1, together with either pD-mCherry or pD-mCherry-**sg**. The percentages of mCherry^+^ cells represent the HDR efficiencies. **c** Effects of HA length of conventional and double cut donors on HDR efficiency. n = 3 biological replicates; *error bars* represent S.E.M. Significance was calculated using the Student’s paired t-test: *P ≤ 0.05; **P ≤ 0.01; ***P ≤ 0.001; *ns* not significant

For conventional HDR-mediated gene targeting, many investigators use HA lengths in the range of 0.1–2 kb for transgene insertions when a DSB is induced at the insertion site [20, 33, 35–42]. Having demonstrated the superior performance of double cut HDR donor in HDR, we decided to examine the effects of HA length on HDR efficiency. To this purpose, we designed a series of donors with HA in the range of 50–1500 bp in length. All of the double cut donors contain target sequence of sgRNA1 to flank the donor plasmids and can be linearized inside cells after co-transfection with Cas9 and sgRNA1 (Fig. 2a). As a control, we also designed a series of conventional circular HDR donors with various HA in the range of 300–1500 bp. We did not construct circular donors with shorter HA because HDR efficiency was as low as 0.22% when HA is 300 bp (Fig. 2b, c and Additional file 1: Figure S1). When HA of the circular donors increased from 300 bp through 600–900 bp, HDR efficiency increased to 10% (Fig. 2b, c and Additional file 1: Figure S1).

Double cut donors increase the events of NHEJ [26], thus the donor with 0 bp HA (pD-mCherry-sg-HA0-0 bp) was constructed to control the events of NHEJ. When 293 T cells were transfected with this donor, only 0.6% of cells expressed mCherry (mCherry^+^), suggesting that NHEJ contributes only minimally to the percentage of mCherry^+^ cells (Fig. 2b and Additional file 1: Figure S1). This result validates the use of percentage of mCherry^+^ cells as an indicator of HDR efficiency. The HA as short as 50 bp led to a 6–10% HDR efficiency. With the increase of HA from 50 bp through 100–150 bp, a twofold increase in HDR efficiency was observed, suggesting that optimal HA length is at least 150 bp. A further increase of HA in double cut donors led to a gradual increase of HDR efficiency to 26% (Fig. 2b, c and Additional file 1: Figure S1).

Taken together, the above results conducted in 293 T cells suggest that a short HA of 300 bp in circular donor is inefficient for HDR, whereas the same HA in double cut donor leads to significant HDR. The double cut donor system not only increases the HDR efficiency, but also reduces the demand for HA length.

### Enhanced HDR editing at the *CTNNB1* locus in iPSCs with double cut HDR donors

**Fig. 3:**
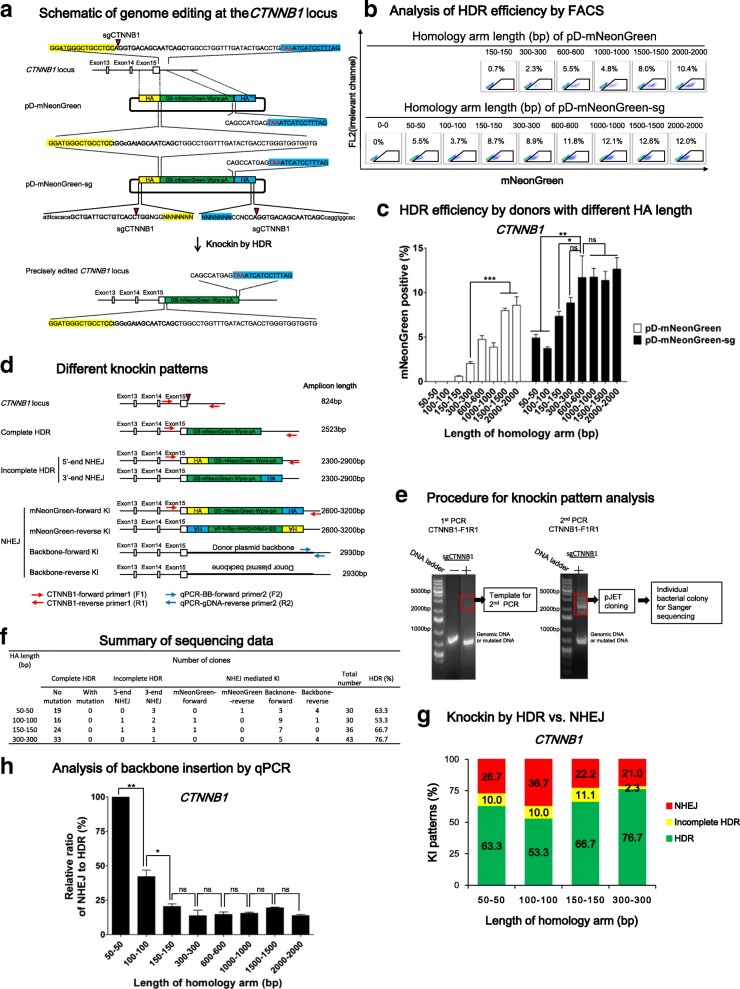
Genome editing in iPSCs at the *CTNNB1* locus with conventional vs. double cut HDR donors of 50–2000 bp in HA length. **a** *Schematic* of genome editing at the *CTNNB1* locus. The double strand break (DSB) is created by Cas9/sgCTNNB1 39 bp before the stop codon TAA (marked in *red*). The donors contain a GS-mNeonGreen-Wpre-polyA sequence sandwiched by left HA (*yellow shadow*) and right HA (*blue shadow*). GS is a linker. Silent mutations (*lowercase* and *bold*) were introduced to prevent cleavage by Cas9/sgCTNNB1. sg*CTNNB1* sequence: *bold*; cut site: *green triangle*; stop codon: *red*; backbone: *lowercase*. **b** FACS analysis of iPSCs three days after nucleofection. The percentages of mNeonGreen^+^ cells represent the HDR efficiencies. **c** Effects of HA length of conventional and double cut donors on HDR efficiency at the *CTNNB1* locus. n = 4. **d** *Schematic* of different knockin patterns. Apart from being edited by HDR, linearized insert sequence or backbone sequence can also integrate into the locus through incomplete HDR (HDR at one side and NHEJ at the other side) or NHEJ. A pair of primers (*red arrows*) was used to amplify edited sequence. The amplicon size is shown at the right side. For NHEJ knockin patterns, the length of PCR product is imprecise because NHEJ might be accompanied by indels. **e** Procedure for knockin pattern analysis. PCR was carried out for twice. The bands between the 2000–4000 bp area were cut off and cloned into pJET vector and individual bacterial colonies were picked for Sanger sequencing. **f** Summary of Sanger sequencing results. **g** Distribution of different knockin patterns by double cut HDR donors with different HA lengths. **h** Quantitative PCR (qPCR) analysis of donor plasmid backbone-forward insertion. *y-axis* indicates the relative ratio of NHEJ/HDR, in which NHEJ was calculated by qPCR data and HDR by the percentage of mNeonGreen^+^ cells in a certain sample. Primers (F2 and R2) for qPCR analysis are indicated in *blue* in (**d**). n = 3. **c**, **h** *Error bars* represent S.E.M. *P ≤ 0.05; **P ≤ 0.01; *ns* not significant, by Student’s paired t-test

With promising results obtained in the 293 T reporter system, we attempted to edit a human iPSC line [43], because of its significance in regenerative medicine and well-known difficulty in editing human iPSCs in comparison to 293 T cells [26]. We first chose to target *CTNNB1*, a pivotal gene in the canonical WNT pathway that is constitutively expressed in iPSCs and other cells. We used a sgCTNNB1 to target 39 bp before the stop codon (Fig. 3a), which showed a 60% cleavage efficiency in iPSCs (Additional file 1: Figure S2). We then constructed a series of donors with GS-mNeonGreen-Wpre-polyA sequence being flanked by HA to this locus on both sides with various lengths (Fig. 3a, b). Silent mutations inside the gene were introduced to prevent cleavage in the middle of the donor by sgCTNNB1. GS is a quadruple GGGGS linker and mNeonGreen is a bright fluorescent protein [44]. HDR-mediated knockin leads to the formation of a CTNNB1-mNeonGreen fusion protein that fluoresces green. Similar to the above design, we constructed a series of circular donors (pD-mNeonGreen) with HA in the range of 150–2000 bp and double cut donors (pD-mNeonGreen-sg) with HA of 50–2000 bp (Fig. 3b).

As the HA length of pD-mNeonGreen donors increased from 150 bp to 2000 bp, HDR efficiency at *CTNNB1* progressively increased from 0.7% to 11% (Fig. 3b, c). In comparison, pD-mNeonGreen-**sg** donors showed 4–5% HDR efficiency even with short HA of 50–100 bp. An increase of HA from 150 bp through 300–600 bp led a gradual increase of HDR from 8% to 12%. However, further elongation of HA to 1000 bp, 1500 bp, or 2000 bp in the double cut donors did not significantly increase HDR efficiency (Fig. 3b, c). Consistent with the notion that homologous recombination depends on HA, a donor with 0 bp HA (pD-mCherry-sg-HA0-0 bp) showed 0% HDR efficiency (Fig. 3b).

As double cut HDR donors have been used for NHEJ-mediated knockin [26], we determined NHEJ insertion events in this highly efficient genome editing system. Besides precise editing by HDR, there are two major possibilities of partial HDR and four possibilities of NHEJ insertions (Fig. 3d). To investigate these events in bulk iPSCs, we designed a pair of primers to specifically amplify the genomic locus without amplifying donors with HA of 50 bp, 100 bp, 150 bp, or 300 bp (Additional file 1: Figure S3). The expected amplicon length is 824 bp for wild-type allele and 2000–4000 bp for the edited allele (Fig. 3d). As expected, the first PCR gave a dominant 824 bp band and weak bands of 2000–4000 bp, which were cut out for a second PCR. We purified and cloned the PCR product in the size of 2000–4000 bp into pJET vector and picked single bacterial colonies for Sanger sequencing (Fig. 3e).

We analyzed sequencing results from at least 30 clones per donor with quality data at both ends (Fig. 3f, Additional file 1: Figure S3). The majority of knockin events were HDR, with a 77% precise insertion rate being observed with HA of 300 bp (Fig. 3f, g). Due to limited numbers of clones that can be picked for Sanger sequencing, we decided to quantitate the relative NHEJ events by quantitative PCR (qPCR). We detected multiple forward backbone insertion (Fig. 3f), which can be used as a surrogate indicator to assess NHEJ. Primers were designed to specifically amplify this particular NHEJ event (Fig. 3d). The relative ratio of NHEJ/HDR was calculated by qPCR data (which is designed to amplify NHEJ insertion of plasmid backbone) divided by percentage of mNeonGreen^+^ cells (which reflects HDR insertion) in a certain sample. With the increase of HA from 50 bp to 300 bp, the relative NHEJ was significantly dropped by 80%, whereas further increase of the HA length did not lead to a further decrease in NHEJ (Fig. 3 h), suggesting that a 300 bp homology on both arms of double cut donors is sufficient to increase HDR and/or suppress NHEJ.

### Reducing the length of replaced sequence surrounding DSB site enhances HDR

**Fig. 4:**
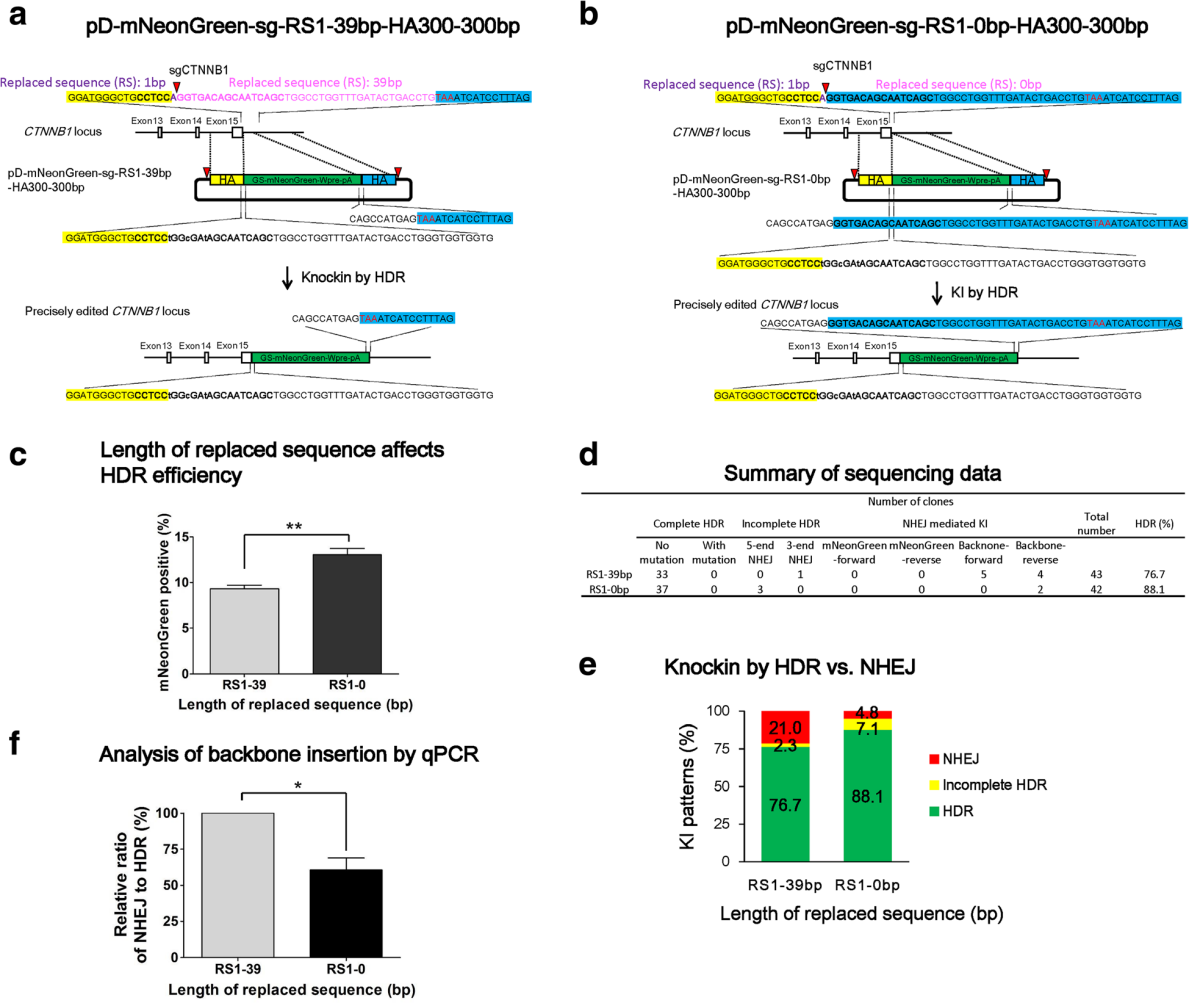
Reducing the length of replaced sequence surrounding DSB improves HDR and reduces NHEJ. **a** *Schematic illustration* of the replaced sequence (RS) in pD-mNEonGreen-sg-**RS1-39 bp**-HA300-300 bp (same as pD-mNEonGreen-HA300- 300 bp) donor. Before integration of the insert by HDR, 1 bp in the left arm (in *purple*) and 39 bp on the right arm (in *pink*) need to be replaced. **b** *Schematic illustration* of the replaced sequence in pD-mNEonGreen-sg-**RS1-0 bp**-HA300-300 bp. Compared to the former donor, the homology in the right arm in this donor extends to the cut site on genomic DNA, making the replaced sequence to be 0 bp on the right side. **c** Effects of RS length on HDR efficiency. n = 3; *error bars* represent S.E.M. **P ≤0.01, by Student’s paired t-test. **d** Summary of Sanger sequencing results. **e** Distribution of different knockin patterns when using the two donors. **f** qPCR analysis of donor plasmid backbone-forward insertion. n = 3; *error bars* represent S.E.M. *P ≤ 0.05, by Student’s paired t-test

The above study has achieved 77% precise editing in more than 10% of iPSCs. This HDR rate at the *CTNNB1* site is lower than the *PRDM14* site (see below). We noticed that, in the above donor design, sequences surrounding the Cas9/sgCTNNB1 cut site, 1 bp in the left arm and 39 bp on the right arm, need to be replaced before the sequence between the two HA on the donor can be inserted (Fig. 4a). After DSB formed, the sequence surrounding DSB will be used to search for the homologous sequence, thus the replaced sequence may be detrimental to HDR. We thus hypothesize that decreasing the length of replaced sequence will increase HDR and decrease NHEJ.

To test this hypothesis, we constructed another pD-mNeonGreen-sg of 300 bp homology. Compared to the former pD-mNeonGreen-sg-**RS1-39 bp**-HA300-300 bp (same as the donor pD-mNeonGreen-HA300-300 bp), the right HA in the new donor pD-mNeonGreen-sg-**RS1-0 bp**-HA300-300 bp extends to the cut site on genomic DNA, making the replaced sequence (RS) to be 0 bp on the right side (Fig. 4a, b). As expected, the decrease of RS from 1-39 bp to 1-0 bp led to a 45% improvement in HDR efficiency (Fig. 4c), and knockin pattern analysis showed that the proportion of NHEJ decreased from 21% to 5% (*P* < 0.05). Consequently, the HDR occurrence in bulk population increased from 77% to 88% (Fig. 4d, e, Additional file 1: Figure S4). In agreement with this result, qPCR that examines backbone insertion indicated a ~40% decrease in the NHEJ/HDR ratio (*P* < 0.05) (Fig. 4f).

To further validate this finding, we used 293 T reporter lines engineered with either a 50 bp or 200 bp sequence that need to be replaced on one or two arms before HDR. As expected, HDR rate was significantly decreased with RS of 200 bp in one arm. When RS was present on both arms, an up to 50% decrease in HDR was observed (Additional file 1: Figure S5). Taken together, these results suggest that in order to achieve high-level HDR and minimize NHEJ, two HA of the double cut donors should be identical to the sequences surrounding DSB.

### High HDR efficiency and low NHEJ occurrence at the *PRDM14* locus by double cut HDR donor with short homology arms

**Fig. 5:**
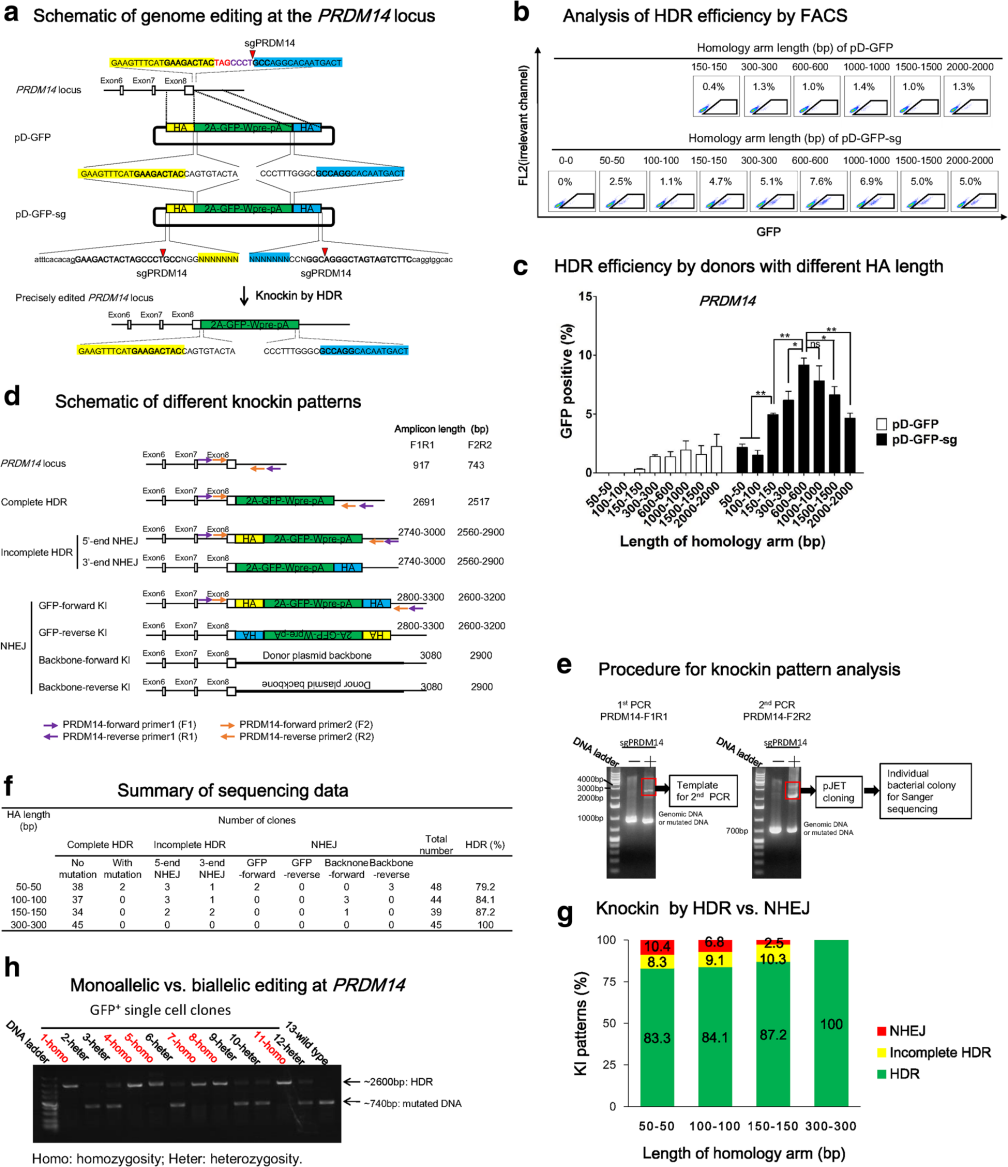
High HDR efficiency at the *PRDM14* locus is achieved by double cut HDR donor with short HA. **a** *Schematic* of genome editing at the *PRDM14* locus. An sgPRDM14 was designed to create DSB at 4 bp after the stop codon TAG (marked in *red*). The donors contain a 2A-GFP-Wpre-polyA sequence sandwiched by left HA (marked in *yellow shadow*) and right HA in *blue shadow*. The PRDM14 and GFP open reading frames (ORF) are fused by a ribosome skipping sequence 2A. pD-GFP is a conventional circular HDR donor and pD-GFP-sg is a double cut HDR donor flanked with the sgPRDM14 target sequence. sgPRDM14 sequence: *bold*; cut site: *red triangle*; plasmid backbone: *lowercase*. **b** Determination of HDR by FACS. iPSCs were analyzed three days after nucleofection of Cas9, sgPRDM14 together with either pD-GFP or pD-GFP-sg. The percentages of GFP^+^ cells represent the HDR efficiencies. **c** Effects of HA length of conventional and double cut donors on HDR efficiency at the *PRDM14* locus. n = 4; *error bars* represent S.E.M. *P ≤ 0.05; **P ≤ 0.01; ***P ≤ 0.001; *ns* not significant, by Student’s paired t-test. **d** *Schematic* of different knockin patterns. Apart from being edited by HDR, cells could also be integrated with linearized inert sequence or backbone sequence through incomplete HDR or NHEJ. Two pairs of primers were designed to amplify the edited locus. The amplicon size is listed at the *right side*. **e** Procedure for knockin pattern analysis. PCR was carried out twice. The bands between the 2000–4000 bp area were cut off and cloned into pJET vector and individual bacterial colonies were picked for Sanger sequencing. **f** Summary of Sanger sequencing results. **g** Distribution of different knockin patterns by double cut HDR donors. **h** Determination of monoallelic vs. biallelic HDR by donor pD-GFP-sg-HA300-300 bp. Twelve edited clones were analyzed

Due to heterogeneity of each locus, we asked whether the above results can be replicated in another site. We applied the same experimental strategy to another gene, *PRDM14*, a regulator of pluripotency [45]. An sgPRDM14 was designed to target the sequence surrounding the stop codon, with cleavage site at 4 bp downstream of the stop codon, and the cleavage efficiency of this sgPRDM14 was ~30% in iPSCs (Additional file 1: Figure S2). We designed donors to in-frame insert 2A-GFP-Wpre-ployA sequence before the stop codon, in which the 2A ribosome-skipping sequence allows for co-translation of PRDM14 and GFP. Similar to CTNNB1, we designed a series of circular HDR donors and double cut HDR donors with various HA length (Fig. 5a). A donor with 0 bp HA (pD-mCherry-sg-HA0-0 bp) was used to control the events of NHEJ, which showed a ~0% HDR efficiency (Fig. 5b). FACS analysis showed that HDR efficiencies have a tendency to increase along with the elongation of HA when using pD-GFP, even though the general HDR efficiency was relatively low (1–3%) (Fig. 5b, c). The pD-GFP-sg double cut donors showed a dramatic increase in HDR efficiency when HA length was extended from 50–100 bp (1–3%) to 600 bp (9%) (Fig. 5b, c).

Of interest, we observed that elongation of HA from 600 bp to 1500 bp or 2000 bp led to a considerable decrease in HDR efficiency at the PRDM14 site (Fig. 5c), which is in contrast to the CTNNB1 site (Fig. 3c). This is not an artifact, because the same pattern was observed in independent studies conducted by two experimenters and the identities of the donor plasmids were double-checked. This counterintuitive observation may be unique to this site and would be a subject for future investigation.

Similar to CTNNB1, we designed two pairs of primers to analyze knockin patterns of edited locus by donors with HA of 50 bp, 100 bp, 150 bp, or 300 bp (Fig. 5d–f and Additional file 1: Figure S6). We obtained high quality sequencing data of ~40 clones for each donor plasmid and found that as HA length increased, from 50 bp to 300 bp, the HDR rate increased from 80% to 100% (Fig. 5f, g). These data suggest that high-level HDR can be achieved with double cut donor, at least for locus like PRDM14.

We further examined the occurrence of biallelic HDR in iPSCs. We isolated 12 GFP^+^ single-cell clones by FACS sorting from the sample using pD-GFP-sg-HA300-300 bp as the donor, as this condition provided high HDR efficiency and low NHEJ rate (Fig. 5f, g). PCR analysis showed one (homozygosity) or two (heterozygosity) bands with expected size. The 2600-bp band indicated amplification of HDR-edited allele. Among the random picked 12 clones, 50% showed biallelic HDR editing at the *PRDM14* locus (Fig. 5 h). In all the heterozygous clones, we observed indel mutations (Additional file 1: Figure S7).

### Double cut donor-mediated HDR can be further improved by cell cycle regulators

**Fig. 6:**
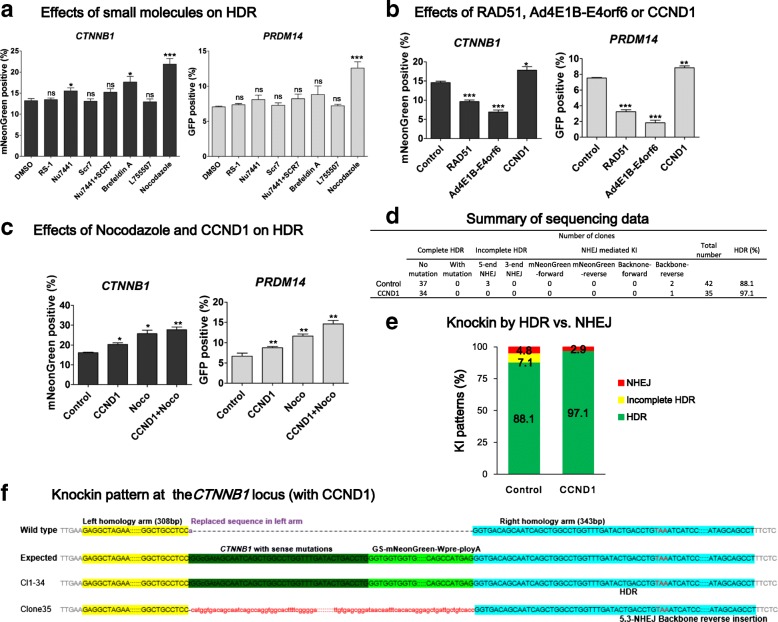
Regulating cell cycle further improves HDR efficiency of the double cut donor system. **a** The effects of small molecules on HDR efficiency at the *CTNNB1* or *PRDM14* locus. The iPSCs were treated with RS-1 (10 μM), Nu7441 (2 μM), SCR7 (1 μM), Brefeldin A (0.1 μM), L755507 (5 μM), or Nocodazole (100 ng/mL) at 0–24 h after nucleofection and the HDR efficiency was determined by FACS on day 3. **b** The effects of RAD51, Ad4E1B-Eorf46, and CCND1 on HDR efficiency at the *CTNNB1* or *PRDM14* locus. The plasmid encoding RAD51, Ad4E1B-Eorf46, or CCND1 was co-transfected with Cas9, sgRNA, and pDonor. The HDR efficiency was examined by FACS on day 3. **c** The effects of Nocodazole and CCND1 on HDR efficiency at the *CTNNB1* or *PRDM14* locus. The plasmid encoding CCND1 was co-transfected with Cas9, sgRNA, and donor plasmid. Nocodazole (100 ng/mL) was added into the medium at 0–24 h after transfection. HDR efficiency was determined by FACS on day 3. **a**–**c** Comparison with control by Student’s paired t-test: *P ≤ 0.05; **P ≤ 0.01; ***P ≤ 0.001; *ns* not significant. **d**–**f** CCND1 increases HDR rate at the *CTNNB1* locus. The procedure for knockin pattern analysis was detailed above. At least 30 colonies were picked for Sanger sequencing at both ends

Several small molecule compounds have been reported to significantly improve the CRISPR-mediated HDR efficiency through different mechanisms [20, 33, 46–48] (summarized in Additional file 1: Table S1). We asked whether these compounds can also increase HDR in our system. We tested multiple small molecules, including RS-1 (a stimulator of human homologous recombination protein RAD51) [49, 50], NU7441 (a DNA-PKcs inhibitor [46]), SCR7 (a DNA ligase IV inhibitor [20, 33, 49]), Brefeldin A [47], L755507 [47, 49], and Nocodazole [48]. To draw a solid conclusion, we examined the effects on HDR of both the *CTNNB1* and *PRDM14* locus using the verified highly efficient pD-sg-HA300-300 bp double cut donor. We added small molecules at their optimal concentration after nucleofection and changed the medium 24 h later. HDR efficiency was analyzed on day 3 by FACS. RS-1, SCR7, and L755507 did not show significant improvement in HDR efficiency at both the PRDM14 and CTNNB loci, while Nu7441 and Brefeldin A showed a less pronounced improvement only at the *CTNNB* locus (*P* < 0.05). In contrast, treatment with Nocodazole, which synchronizes cell cycle at G2/M phase, increased HDR efficiency by ~50% at both loci (*P* < 0.001) (Fig. 6a). Similar results were observed in H1 the human ES cell line (Additional file 1: Figure S8), demonstrating the reproducibility of this finding.

We also examined the effects of overexpression of RAD51, a key factor in the homologous recombination pathway, and Ad4E1B-E4orf6, which were reported to considerably increase HDR by inhibiting NHEJ [51]. In contrast to expectations, RAD51 or Ad4E1B-E4orf6 significantly decreased HDR efficiency in our system (*P* < 0.001), at the two loci (Fig. 6b). Encouraged by the HDR-enhancing effects of Nocodazole, we tested CCND1, also known as cyclin D1, which induces cell cycle transition from G0/G1 to S-phase [52]. CCND1 showed a ~20% improvement in HDR at both sites (Fig. 6b). We further applied Nocodazole and CCND1 together and found that they had an additive effect and increased HDR efficiency by 80–100% at both loci (Fig. 6c). This observation may be explained by that the combined use of CCND1 and Nocodazole increases cells in S/G2/M phases during which HDR is efficient, while they considerably decrease cells in G0/G1 phase during which double-stranded DNA (dsDNA) breaks are predominated repaired by NHEJ. As such, Nocodazole and CCND1 have an additive effect on enhancing precise genome editing.

Last, we asked whether increased HDR by CCND1 may lead to decreased NHEJ. We only tested the *CTNNB1* locus, as using a 300 bp homology at *PRDM14* already decreased NHEJ to a very low level. We used the optimized double cut donor pD-mNeonGreen-sg-RS1-0 bp-HA300-300 bp, which still showed an appreciable NHEJ rate. However, after co-transfection of iPSCs with CCND1 and HDR plasmids, the NHEJ events were decreased from 12% to 3% (Fig. 6d–f). These data suggest that cell cycle regulators not only increase HDR but also suppress NHEJ.

High HDR efficiency in human iPSCs using this approach will find its way to diverse applications. Take one, after tagging a gene of interest with a flexible G4S linker and a fluorescent protein gene, a fusion protein will be formed, which can be used to quantitate the gene expression level by FACS and map its cellular localization by confocal microscopy. Here we targeted *mNeonGreen* to the stop codon of *CTNNB1* and *tdTomato* to the stop codon of *PRDM14* and examined the cells by confocal microscopy. As expected, the PRDM14-tdTomato fusion protein was localized in the nucleus (Additional file 1: Figure S9a). Of interest, we observed the mNeonGreen signal on plasma membrane, indicating that CTNNB1 is largely localized at iPSC membrane (Additional file 1: Figure S9b).

## Discussion

Here we report that a double cut donor vector design together with the cell cycle regulators leads to high-level HDR-mediated precise integration of a 2-kb piece of DNA in iPSCs. We show that the double cut donor with a targeting cassette flanked by two sgRNA recognition sequences leads to a twofold to fivefold increase in HDR efficiency compared to the circular donor plasmid. The optimal HA length of double cut donors for achieving high-level HDR is 600 bp and further elongation only slightly increases or even decreases HDR at different sites. Minimizing the replaced sequence surrounding the DSB increases HDR and suppresses NHEJ-mediated insertion. The combined use of cell cycle regulators Nocodazole and CCND1 leads to an additional 100% increase in HDR efficiency. After optimization, less than 3% NHEJ-mediated insertion of donor plasmid sequence can be detected. These findings should have important implications for precision medicine.

The length of HA is the most critical factor for targeting template design. For precise editing of point mutations or SNPs, ssODNs are often used as a DNA template and homology of 30–70 bases on either arm is sufficient for high-level HDR [30, 53]. However, precise editing, in particular insertion of a large piece, remains inefficient in cells of significant research and clinical interest, such as human pluripotent stem cells. In early gene targeting studies, the homology of the two arms was recommended to be 5–8 kb in total and more than 1 kb for the shorter arm. After the advent of artificial nuclease and CRISPR technologies, HA lengths ranging from 100 bp to several kbp have been used for successful gene editing [20, 33, 40–42]. The optimal HA length is reported to be 2 kb, at least in human iPSCs [20]. We reasoned that simultaneous cleavage of the genomic and plasmid DNA makes the HA more accessible, thus the optimal HA might be shorter. To identify the optimal HA length, we evaluated the homology length in the range of 50–2000 bp at two to three distinct loci. The shortest length we tested is 50 bp, because 40–50 bp HA have been previously used by other investigators [27, 34, 54].

Using stringent promoterless donor HDR reporter systems, we systematically compared conventional circular HDR donors with double cut HDR donors. We constructed donors to insert florescent transgenes into each target locus, so that HDR events can be detected by FACS after precise transgene knockin. Since HDR efficiency differs from one site to another, we studied three sites. We observed that the effects of HA length on HDR efficiency is not identical for the three sites. However, careful examination of our data leads to several conclusions. First, for conventional circular HDR donor, a HA of less than 150 bp is insufficient to guide precise genome editing. An increase of HA from 300 bp to 2000 bp shows a trend of continued increase in HDR efficiency. This is consistent with a previous report showing an optimal HA length of 2000 bp in iPSCs [20]. Second, HA as short as 50 bp in the double cut donor design lead to appreciable HDR, whereas elongation of HA to 150 bp considerably increases HDR efficiency. The highest HDR is achieved when HA is 600–1000 bp in length. Third, the use of double cut donor increases HDR efficiency by twofold to fivefold compared to circular plasmid donor. We noted that the optimal HA length is very similar to the DNA length dependence of the single-strand annealing (SSA) pathway in *Saccharomyces cerevisiae*, the budding yeast [55], suggesting that the DSB in our system is most probably repaired by the SSA pathway. This may also explain why most HA enhancers and NHEJ inhibitors do not show positive effects on HDR in our study.

Taking all factors into consideration, we recommend the use of 600 bp HA for double cut donor vector design, for the following reasons. First, 600 bp is the optimal length for some sites and further increases in HA length only slightly enhances HDR at some locus, while a HA with more than 1000 bp may be even detrimental for some locus. Second, construction of donor vector with two 600 bp homology arms is an economical choice, because PCR amplification of short pieces from the genomic DNA (gDNA) template is very efficient. In addition, the vector verification can be easily conducted by Sanger sequencing, which usually provides quality data of 700–800 bp.

In our study, we used one sgRNA to target both gDNA and the donor plasmid. In previous studies, two sgRNAs were used, one for creating genomic DSB and another for releasing donor template from the plasmid [26, 27]. This design increases complexity and occasionally may not be able to perfectly synchronize the demand and supply of homologous sequences, because the cleavage efficiencies of the two distinct sgRNAs may not be identical. In support of this hypothesis, we found that the use of two sgRNAs in the double cut donor system decreases HDR by ~10%, albeit no significant difference is achieved (Additional file 1: Figure S10).

Another consideration for donor template design is the distance of the insertion locus from the DSB, which should be as short as possible and 100–200 bp away, might decrease the knockin efficiency by ~80% when a conventional donor plasmid is used [37]. In the double cut donor system, a 50–100 bp replaced sequence leads to a 50% decrease in HDR efficiency in 293 T cells (Additional file 1: Figure S5). Similarly, a 39 bp replaced sequence at the *CTNNB1* locus induces a 30% decrease in HDR in iPSCs (Fig. 4c). As such, the ideal donor should be designed to have two HA identical to sequences surrounding DSB created by Cas9/sgRNA.

We also investigated whether commonly used HDR boosters can improve knockin efficiency in our system. Thus far, several compounds have been reported to be able to improve knockin efficiency. Suppression of the NHEJ key enzymes such as DNA Ligase or DNA PK has been shown to stimulate Cas9-mediated HDR at the expense of NHEJ [20, 33, 46]. NU7441, an inhibitor of DNA-PKcs, increases the knockin efficiency by twofold in 293 T cells [46]. SCR7, an inhibitor of DNA ligase IV, improves the knockin efficiency by twofold to 19-fold in two reports [20, 33]. Brefeldin A and L755507 increase HDR twofold to threefold in mouse pluripotent stem cells [47]. In addition, RS-1, which upregulates homologous recombination factor RAD51 [56], increases nuclease-mediated knockin efficiency by twofold to fivefold [49, 50]. In contrast to these reports, none of these molecules consistently enhances HDRs at both *CTNNB1* and *PRDM14* loci in human iPSCs. In further support of these results, we found that overexpression of RAD51 or Ad4E1B-E4orf6, which degrade ligase IV, is detrimental to HDR (Fig. 6b). One explanation for the discrepancy between our results and the published data is that our optimized vector has achieved high-level HDR, which masks the subtle changes mediated by many inhibitors. Alternatively, the effects of these factors are cell line dependent. In support of this notion, unimpressive effects of SCR7 and L755507 were also reported in recent publications using different cell lines or cells from different species [49, 50].

Another strategy to enhance HDR takes advantage of the fact that NHEJ occurs during the G1, S, and G2 phases, whereas HDR-mediated repair is restricted to the late S and G2 phases [57, 58]. Timed delivery of Cas9 protein and sgRNAs into cells arrested at the G2/M phase after Nocodazole treatment significantly increases HDR events [48]. We found that cell cycle synchronization with Nocodazole in our plasmid Cas9/sgRNA system can increase HDR efficiency by ~50% (Fig. 6a and Additional file 1: Figure S8). In addition, CCND1 increases HDR by ~20%, which may take effect by pushing cells at G1 phase into the S phase. Moreover, Nocodazole synergizes with CCND1 to increase HDR efficiency by ~100%. The additive effect is due to Nocodazole synchronizing the cell cycle at the G2/M phase and to CCND1 inducing cell cycle transition from the G0/G1 to the S phase, leading to more cells in the S/G2/M phases during which dsDNA breaks are preferentially repaired by HDR instead of NHEJ. Similar strategies have been used to increase HDR and decrease NHEJ incidence by creating a fusion protein of Cas9 and a peptide from Geminin, which is expressed during S/G2/M and degraded during G1 [59, 60]. However, this approach may occasionally decrease HDR due to decreased Cas9 protein levels. The use of CCND1 is preferable, because it enhances HDR as well as decreases NHEJ insertion. Alternatively, integration of all these strategies may lead to enhanced precise genome editing.

HDR efficiency in human pluripotent stem cells, including iPSCs or embryonic stem cells, is often much lower than other types of cells [61]. Approximately 1% or lower HDR efficiency is generally achieved after inducing DSB [21, 42, 62, 63], although an up to 11% efficiency has been reported, which might be cell line dependent and is limited to homozygous gene replacement [20]. Using the double cut HDR donor, we achieved ~14% HDR at *CTNNB1* and ~10% at *PRDM14*. Combined use of Nocodazole and CCND1 further increases the HDR efficiency to 20–30%. Given that iPSCs have been widely used for disease modeling and regenerative medicine, this improvement will ease the burden for identification of correctly edited cells by single-cell cloning and sequencing.

Recently, ribonucleoproteins (RNPs), the Cas9 protein in complex with in vitro transcribed guide RNA, have been used together with a single-stranded oligonucleotide HDR template for nucleotide replacement or insertion [48, 64, 65]. Moreover, chemically modified guide RNAs can further enhance genome editing efficiency [66]. We believe that the double cut donor vector can also be delivered together with Cas9 protein/RNA and sgRNA transcripts, which may even be preferable in applications such as zygote injection for creating knockin animals.

## Conclusion

In summary, synchronizing the demand and supply of homologous sequences, by flanking the targeting vector with two sgRNA recognition sites that are identical to the sgRNA target site on the genome, leads to a twofold to fivefold increase in HDR-mediated knockin. The double cut donor template design requires HA as short as 600 bp to enable high-level precise insertion of a large piece of DNA. We believe that the improved targeting strategies are broadly applicable in generating precise knockin or reporter animals and human cell lines for basic research and disease modeling. Further improvements of the double cut donor system may contribute to the success of next-generation clinical gene therapy.

## Methods

### Lentiviral vector construction

The complementary DNA (cDNA) for a puromycin resistant gene (Puro) was amplified by PCR and purified using KAPA HiFi polymerase (KAPA Biosystems) and a GeneJET Gel Extraction Kit (Thermo Fisher Scientific), respectively. The open reading frame of the Puro gene was inserted into a lentiviral vector with the EF1 promoter, used to drive the expression of Puro. The sgGFP target sequence together with the NGG PAM (GGTGCAGATGAACTTCAGGG) was in-frame PCR-cloned into the vector by incorporating the relevant sequences in the primers. To construct vectors with the GFP gene between the Puro and Wpre element, while still harboring sequences surrounding DSB that are non-homologous to the targeting vector, a ~50 bp or ~200 bp irrelevant sequence was introduced between the sgRNA1 recognition sequence and the Puro and/or Wpre element. Multiple gene inserts were cloned into lentiviral vector backbones using the NEBuilder HiFi DNA Assembly Kit (New England Biolabs), following manufacturer’s instructions. All constructs were verified by Sanger sequencing (MCLAB). Correct clones were grown in CircleGrow Media (MP Biomedicals) and DNA plasmids were purified using Endo-Free Plasmid Maxi Kits (Qiagen). A standard calcium phosphate precipitation protocol was used for lentivirus production as previously described [32]. The lentiviral vectors were concentrated a 100-fold by centrifugation at 6000 g for 24 h at 4 °C to reach titers of 2–10 × 10^7^/mL.

### GFP reporter cell line

Human embryonic kidney (HEK) 293 T cells were transduced with lentiviral vectors (Lenti-EF1-Puro-GFP-Wpre, Lenti EF1-Puro-sgRNA1-Wpre; Lenti EF1-Puro-GFP(nh0-200 bp)-Wpre, Lenti EF1-Puro-GFP(nh200-0 bp)-Wpre, Lenti EF1-Puro-GFP(nh50-50 bp)-Wpre, and Lenti EF1-Puro-GFP(nh200-200 bp)-Wpre) at a low MOI of 0.1–0.2, and stably transduced cells were selected for by supplementing culture medium with 1 μg/mL puromycin. After one week of antibiotic selection, cell lines expressing puromycin resistance and either high GFP (>98%) or no GFP production (in cases of GFP fragments or no GFP insert) were established.

### sgRNA design

The CHOPCHOP website (https://chopchop.rc.fas.harvard.edu/) was used to design high-performance sgRNAs targeting various positions on GFP sequence and the stop codons of the human *CTNNB1* and the human *PRDM14* genes [67]. We preferentially chose sgRNAs with a G at the 5′ end which initiates U6-promoter-mediated transcription [16]. Five sgRNAs were used in this study: sgRNA1 (GGTGCAGATGAACTTCA), sgRNA2 (GCTAGTGGGGTTGATAGGAG), sgRNA3 (GCCGGGAGCAGGCGTGAGTG), sgCTNNB1 (GCTGATTGCTGTCACCTGG), sgPRDM14 (GAAGACTACTAGCCCTGCC), among which sgRNA1, sgRNA2, and sgRNA3 do not target human genome.

### Cas9 and sgRNA plasmid construction

All Cas9 and sgRNA plasmids were constructed with a NEBuilder HiFi DNA Assembly Kit (New England Biolabs). First, PCR products were produced using KAPA HiFi polymerase (KAPA Biosystems) and purified using a GeneJET Gel Extraction Kit. The linear PCR products were then assembled into plasmids in a DNA assembly reaction (20 uL), on ice, according to the manufacturer’s instructions. The reaction contained NEBuilder HiFi DNA Assembly Master Mix (10 uL), equal ratios of PCR products (0.2–0.5pmols), and deionized water. The ligation reaction was briefly vortexed and centrifuged prior to incubation at 50 °C for 5–30 min. NEB 5-alpha Competent *E. coli* cells were then transformed with the assembled DNA products and plated on ampicillin-treated agar plates. Multiple colonies were chosen for Sanger sequencing (MCLAB) to identify the correct clones using the primer U6-F: GGGCAGGAAGAGGGCCTAT.

### Donor plasmid construction

All of the donor plasmids used in this study were generated with a CloneJET PCR Cloning Kit (Thermo Scientific). To construct pJET donor plasmids, the homology repair templates were amplified by PCR using KAPA HiFi polymerase (KAPA Biosystems) and purified using a GeneJET Gel Extraction Kit. To clone donor plasmids harboring sgRNA recognition sites, the sgRNA target sequence together with a PAM (NGG) was included in both the forward and the reverse primers. A ligation reaction (20 uL) was performed, on ice, according to the manufacturer’s instructions, containing 2X Reaction Buffer (10 uL), pJET1.2/blunt Cloning Vector (50 ng/μL) (1 uL), T4 DNA Ligase (1 uL), purified PCR product (0.15 pmol), and nuclease-free water (remaining volume). The ligation reaction was then briefly vortexed and centrifuged prior to incubation at room temperature (22 °C) for 5–30 min. NEB 5-alpha Competent *E. coli* cells were then transformed with the ligation product and plated on ampicillin-treated agar plates. Multiple colonies were chosen for Sanger sequencing (MCLAB) to identify the correct clones using the primers pJET1.2-F: CGACTCACTATAGGGAGAGCGGC and pJET1.2-R: AAGAACATCGATTTTCCATGGCAG. Correct clones were cultured and DNA plasmids were purified, as previously described. To construct donor plasmid targeting *CTNNB1* at 39 bp before the stop codon, the left and right HA were amplified from human genomic DNA; with the stop codon being removed and in-frame linked with the GS sequence (a quadruple of GGGGS peptides); the insert mNeonGreen-Wpre-polyA was amplified from another vector in the lab. A sg*CTNNB1* target sequence together with the PAM sequence (GCTGATTGCTGTCACCTGGAGG) was tagged at a location outside of the two HA. To construct donor plasmids targeting the *PRDM14* stop codon, the left and right HA were amplified from human genomic DNA, with the stop codon being removed and in-frame linked with the 2A sequence; the insert 2A-GFP-Wpre-polyA was amplified from another vector in the lab. A sg*PRDM14* target sequence together with the PAM sequence (GGAAGACTACTAGCCCTGCCAGG) was tagged to the regions flanking the upstream and downstream HA. The *CTNNB1* and *PRDM14* donor template plasmids were generated with a NEBuilder HiFi DNA Assembly kit (New England Biolabs), as previously described.

### Construction of putative HDR-enhancing plasmids

To test if extra factors can enhance double cut donor mediated HDR, we examined RAD51, CCND1 (Cyclin D1), and Ad4 E1B55K and E4orf6 [51]; the latter two genes were linked together using a ribosome-skipping E2A sequence. cDNAs for RAD51 and CCND1 were purchased from DNASU, and Ad4 E1B and E4orf6 were purchased from Addgene (Plasmid #64218 and 64222). The EF1 promoter was used to drive the expression of these genes and the Wpre-polyA cassette was tagged downstream of the stop codon to increase transgene expression levels. All vectors were confirmed by sequencing.

### Cell culture

HEK 293 T cells were cultured in DMEM (Dulbecco’s modified Eagle medium) supplemented with 10% fetal bovine serum (FBS; ABM) and 1% penicillin/streptomycin. Human iPSCs used in this study were generated from peripheral blood mononuclear cells after lentiviral transduction of five reprogramming factors [43]. Feeder-free iPSCs were maintained on Matrigel-coated plates (BD) and cultured in mTeSR1 (Stemcell Technologies) according to the manufacturer’s instructions. The ROCK inhibitor Y-27632 (10 μM, Millipore) was added to the culture medium before, during, and after, passaging with Accutase. All cells were cultured at 37 °C with 5% CO_2_.

### Transfections

For transfection of HEK 293 T cells, Lipofectamine 3000 (Life Technologies) was used according to manufacturer’s instructions. For transfection of human iPSCs, cells were electroporated using the Lonza Nucleofector system (Lonza). The Human Stem Cell Nucleofector® Kit 2 and the program B016 were used as outlined in the manufacturer’s instructions. For nucleofection, 1 μg of each plasmid was used.

### T7E1 cleavage assay

iPSCs were nucleofected with either sgCTNNB1 or sgPRDM14 together with Cas9 plasmid. Three days later, genomic DNA was isolated using the DNeasy Blood & Tissue Kit (Qiagen) according to the manufacturer’s instructions. For cells targeted with sgCTNNB1, the predicted flanking sequence was PCR-amplified using CTNNB1-F1 and CTNNB1-R1 primers; for cells treated with sgPRDM14, PRDM14-F2 and PRDM14-R2 primers were used. The amplicons were denatured by heating and annealed to form heteroduplex DNA, which was treated with 5 units of T7 endonuclease 1 (New England Biolabs) for 20 min at 37 °C and then analyzed by 2% agarose gel electrophoresis. The cleavage frequency was calculated from the proportion of cut bands intensity to total bands intensity.

### Small molecules

To test the effect of small molecule compounds, iPSCs were evenly split into eight wells after nucleofection with Cas9/sgRNA and the relevant double cut donor. DMSO control (0.1%), RS-1 (10 μM), Nu7441 (2 μM), SCR7 (1 μM), Brefeldin A (0.1 μM), L755507 (5 μM), Nocodazole (100 ng/mL), or Nu7441 (2 μM) and SCR7 (1 μM) were added in the wells for the first 24 h and then the medium was changed with fresh medium thereafter. Three days after nucleofection, cells were harvested for FACS analysis to determine the HDR efficiency in each condition.

### Flow cytometry

To determine the percentage of cells that are mCherry-positive, mNeonGreen-positive, or GFP-positive (Knockin by HDR), cells were disassociated with Accutase and analyzed on a BD FACSAria III flow cytometer. Cells were first gated for the intact cell population using forward scatter versus side scatter plots and then gated for single cells based on forward scatter W versus forward scatter H. The means and standard errors from at least three independent experiments were calculated and statistical significance was determined using the student’s paired t-test.

### Determination of NHEJ-mediated and HDR-mediated knockin by PCR and Sanger sequencing

Human iPSCs were harvested at day 3 after co-transfection of Cas9/sgRNA and donors for DNA extraction. The CTNNB1 and PRDM14 target sequences were amplified with KAPA HiFi DNA polymerase by PCR twice. For the first-round PCR at *CTNNB1* locus, CTNNB1 forward primer-F1 (GTGGCCTGGCACTGAGTAAT) and CTNNB1 reverse primer-R1 (CTCAGCAACTCTACAGGCCA) were used with the PCR cycling condition being 98 °C for 5 min, followed by 98 °C for 5 s, 68 °C for 1 min for 30 cycles. The bands in the size range of 2–4 kb were cut out and purified using the GeneJET Gel Extraction Kit. For second-round PCR, 1 ng of purified primary PCR products was amplified using the same pair of primers and cycling conditions. The bands in the range of 2–4 kb from the second PCR were purified and cloned into the pJET vector. Approximately 50 individual bacterial colonies from each condition were picked for Sanger sequencing. Clones with high-quality sequencing data at both ends were aligned with expected HDR knockin sequence and donor plasmid sequence by BLAST. Clones with wild-type gDNA insertion were reported. Similar procedure was carried out for the *PRDM14* locus with PRDM14 forward primer-F1 (CCAGCCTGCAATCTGCTTTT) and PRDM14 reverse primer-R1 (gccAACTGCAGGGACTTCTA) for the first-round PCR and PRDM14 forward primer-F2 (GACCAGGAGTGCTCTATGGC) and PRDM14 reverse primer-R2 (AGGAAATAGAGAGAATCCGAATCTC) for the second-round PCR. The annealing temperature of 64 °C was used for both first-round and second-round PCR.

### Examination of relative incidence of NHEJ-mediated donor plasmid insertion

To quantitate the occurrence of NHEJ-mediated insertion of large pieces from donor plasmid, we conducted qPCR. We used one possible insertion, backbone-forward insertion, as a surrogate indicator of all the possible NHEJ-mediated insertion of donor plasmid. The real-time PCR reaction system (20 μL) consisted of 10 μL of SYBR Green qPCR Master Mix (2X), 1 μL each of F2 (qPCR-BB-forward primer2, CACTCATTAGGCACCCCAGG) and R2 (qPCR-gDNA-reverse primer2, CCCACCCTACCAACCAAGTC), and 100 ng gDNA from a cell sample from day 3. The cycling conditions were 95 °C for 3 min, followed by 95 °C for 15 s, 64 °C for 20s, and 72 °C for 30s, for 35 cycles. The GAPDH gene, a housekeeping gene, was used to normalize the qPCR data. The relative NHEJ incidence was calculated by the relative value of backbone-forward insertion divided by portion of HDR in the same sample.

### Single-cell cloning and genotyping of edited iPSCs

Human iPSCs were dissociated using Accutase in order to get single-cell suspension. Live iPSCs were suspended in Accutase and did the following single-cell sorting as soon as possible. Single-cell sorted using a BD FACSAria III with a 70 mm nozzle under sterile conditions into 96-well plates coated with Matrigel. Each well contained mTeSR medium as well as the ROCK inhibitor Y-27632 (10 μM). After sorting, plates were cultured at 37 °C with 5% CO2. Colony formation was seen seven days post sorting. The cells were refreshed with mTeSR medium every two to three days.

96-well cells were dissociated using EDTA and transferred into 24-well plates 14 days after FACS. Two or three days later, part of the cells were harvested for DNA extraction using a Genomic DNA Extraction Kit (Qiagen) and the remaining cells were passaged for flow. GFP and PRDM14 sequence was amplified with KAPA HiFi DNA polymerase by PCR using the following primers: PRDM14-HA300KI-F: GACCAGGAGTGCTCTATGGC, PRDM14-HA300KI-R: AGGAAATAGAGAGAATCCGAATCTC. The PCR cycling conditions were 95 °C for 4 min followed by 98 °C for 5 s, 64 °C for 15 s, 72 °C for 3 min, for 35 cycles.

### Confocal microscopy

Cover glass was coated with 0.1% geltin for 30 min at 37 °C and then coated with Matrigel. iPSCs were seeded on cover glass and cultured for two days. Two days later, the cells with mNeonGreen fused in frame to the C-terminus of CTNNB1 were stained with Hoechst 33258 (Sigma) and Cell Mash Deep Red (Life Technology) following the protocol. iPSCs with tdTomato fused in frame to the C-terminus of PRDM14 were fixed with 4% paraformaldehyde (Sigma), followed by staining with Phalloidin 488 (Thermo Fisher Scientific) and DAPI (Sigma). Images were taken using a Zeiss Axio Observer Z1 inverted LSM 710 NLO laser scanning confocal microscope. Different lasers were used to excite each dye. Hoechst 33258 and DAPI were excited with a 405-nm DPSS laser, mNeonGreen and Phalloidin 488 were activated with the 488-nm line of a Kr-Ar multiple laser, and CellMask Red and tdTomato with a 633-nm HeNe laser. For Hoechst 33258 and DAPI, emitted light was collected at 417–679 nm using a PMT array. For mNeonGreen and Phalloidin, emitted light was collected with a single PMT at 493–630 nm. For CellMask Red and tdTomato, emitted light was collected with a single PMT at 638–755 nm.

### Statistics

Data were analyzed by student’s paired t-test for two groups and ANOVA for more than two groups. Clone difference between different groups was analyzed by Fisher’s exact test. All the values were shown as mean ± SEM (standard error of the mean).

## Additional file

Additional file 1: Supplemental Table and Figures. (PDF 5937 kb)
