# Supplementary material for: Efficient precise knockin with a double cut HDR donor after CRISPR/Cas9-mediated double-stranded DNA cleavage
Source: Genome Biol. 2017 Feb 20;18:35. doi: 10.1186/s13059-017-1164-8 (PMC5319046; doi:10.1186/s13059-017-1164-8)
Supplement: Additional file 1: — Supplemental Table and Figures. (PDF 5937 kb) [file 13059_2017_1164_MOESM1_ESM.pdf]

Supplementary Table 1: Small molecules that are reported to increase HDR efficiency.

Type	Small molecule	Concentration	Time	Cell line	Fold change	Reference	Function
**NHEJ inhibitors**	SCR7	0.01~1μM	24-48h	A549	1.8-3.1	Byrne SM, *et al*, 2015	SCR7 targets the DNA binding domain of DNA ligase IV, reducing its affinity for DSBs and inhibiting its function
		0.01~1μM	24-48h	MelJuSo (melanoma)	10.9-19.1		
		1μM	24-48h	DC2.4 (dendritic cell line)	9.3-14.1		
		1mM	Inject together with Cas9 and sgRNA	Mouse zygote	1.0-4.0		
		1μM	12-72h	HEK293	5	Maruyama T, *et al*, 2015	
		1μM	0-72h	HEK293A	1.2	Pinder J, *et al*, 2015	
		20-80μM	0-20h	Rabbit embryo	NS	Song J, *et al*, 2016	
		1μM	16h-64h	HEK293	2	Robert F, *et al*, 2015	
	NU7441	2μM	16h-64h	HEK293	2		Pharmacological inhibitors of DNA-PKcs
	KU-0060648	250nM	16h-64h	HEK293	2		
**HR activator**	RS-1	10μM	0-72h	HEK293A	3-6	Pinder J, *et al*, 2015	Stabilize association of RAD51 with DNA
		10μM	0-72h	U2OS	1.5-3		
		7.5μM, 15μM	0-20h	Rabbit embryo	2-5	Song J, *et al*, 2016	

Table 1 (Continued)

Type	Small molecule	Concentration	Time	Cell line	Fold change	Reference	Function
**Cell cycle inhibitors**	Lovastatin (Lov)	40μM	0-17h	HEK293T	NS	Lin S, *et al*, 2014	Blocks at early G1 and partially at G2/M phase
				Fibroblast	0.8%		
	Mimosine (Mim)	200μM	0-17h	HEK293T	NS		Arrest cells at the G1-S border prior to onset of DNA replication
				Fibroblast	0.7%		
	Aphidicolin (Aph)	2μg/ml	0-17h,	HEK293T	1.1-1.5		
			25h-42h	Fibroblast	0.6%-1.3%		
	Thymidine (Thy)	5mM	0-17h,	HEK293T	NS		
			25h-42h	Fibroblast	0.6%		
	Hydroxyurea (HU)	2mM	0-17h	HEK293T	NS		
				Fibroblast	0.6%		
	Nocodazole (Noc)	200ng/ml	0-17h	HEK293T	1.5-3		Arrest at G2/M phase
			0-17h	Fibroblast	NS		
			0-16h, Aph 3h	Human ESC	2%		
**Others**	L755507	5μM	0-72h	HEK293A	NS	Pinder J, *et al,* 2015	A β3-adrenergic receptor agonist
	L755507	5μM	0-24h	Mouse ESC	3	Yu C, *et al,* 2015	
	Brefeldin A	0.1μM	0-24h	Mouse ESC	2		An NHEJ inhibitor of intracellular protein transport from the ER to the Golgi apparatus

NS: not significant.

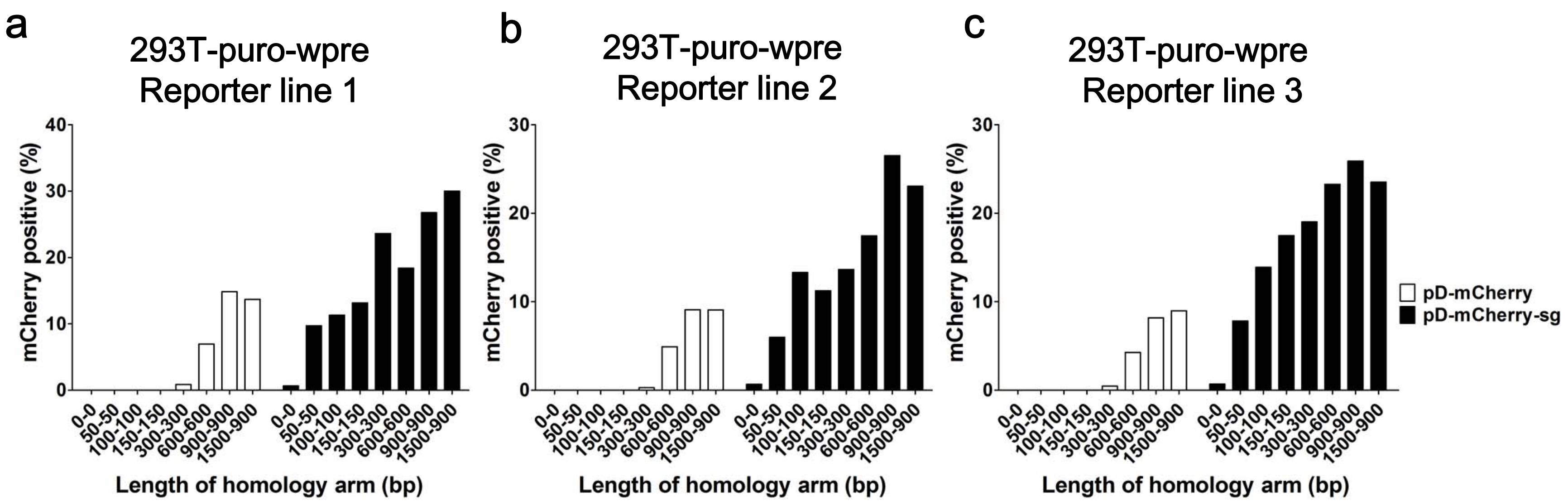

Supplementary Figure 1: The HDR efficiency of different homology arm length in three 293T reporter lines. Single 293T-puro-wpre cell clones 1# (**a**), 2# (**b**) and 3# (**c**) were isolated by sorting. The HDR-mediated knockin efficiencies were determined by FACS at day 7 after co-transfection with Cas9/sgRNA1 and the donor plasmids with various homology arm length. The data presented are average of three technical replicates.

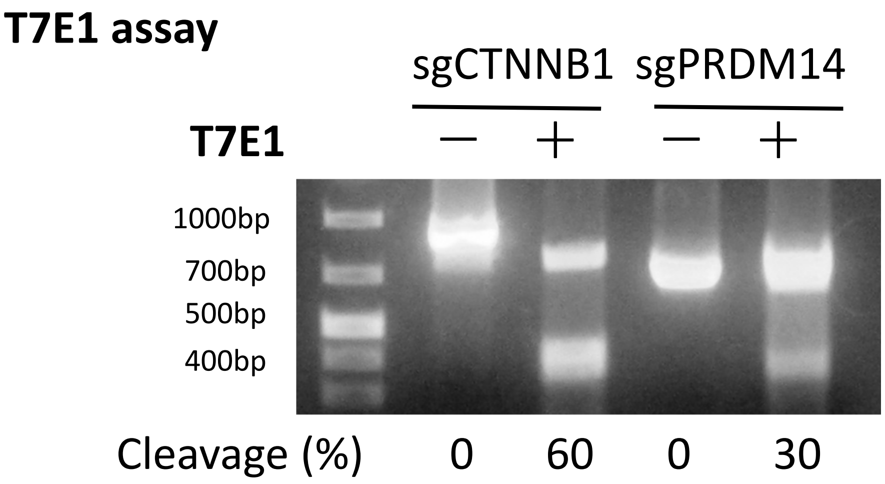

Supplementary Figure 2: The cleavage efficiency of sgCTNNB1 and sgPRDM14 in iPSCs. To confirm the target locus cleavage activity of sgCTNNB1 and sgPRDM14, we nucleofected iPSCs of each sgRNA together with Cas9 plasmid, and carried out T7E1 assay using total genomic DNA. CTNNB1-F1 and CTNNB1-R1 primers, PRDM14-F2 and PRDM14-R2 primers were used for PCR. The cleavage frequency was calculated from the proportion of cut bands intensity to total bands intensity.

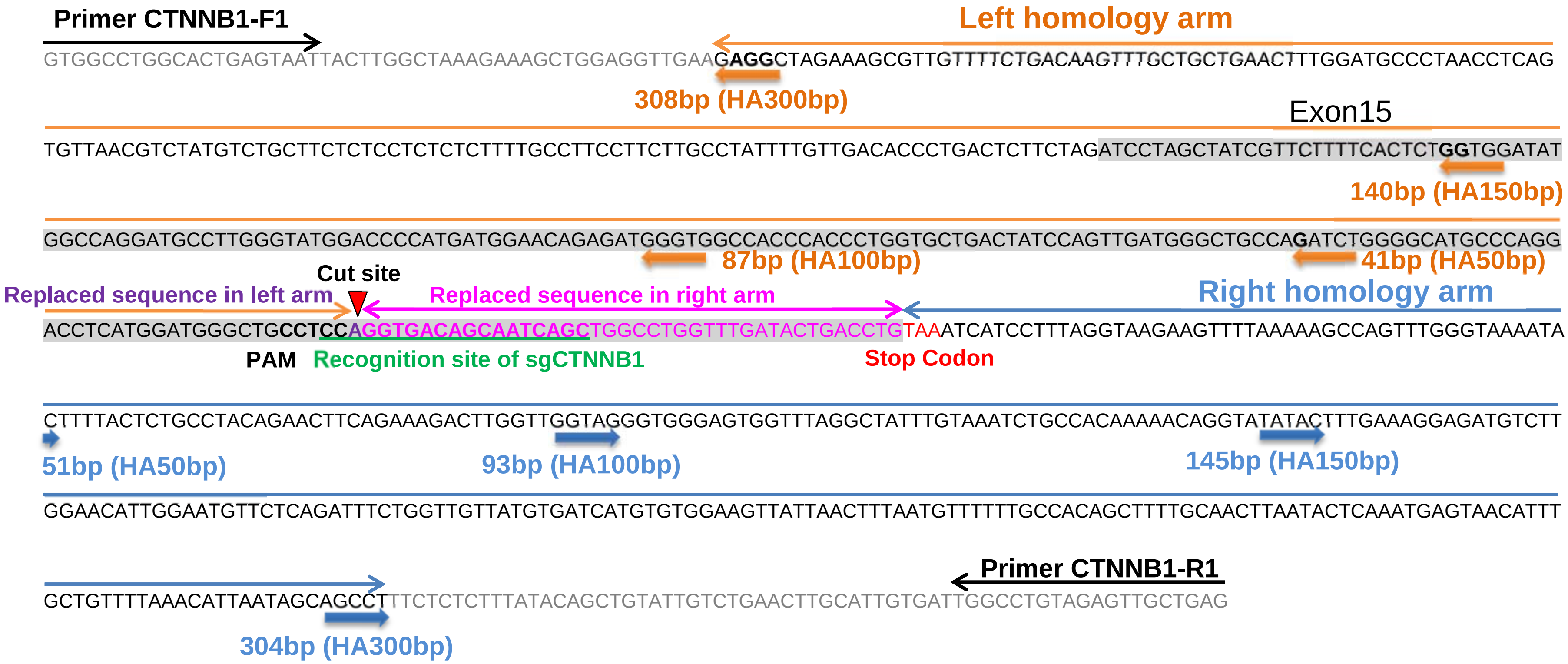

Supplementary Figure 3a: Design of gene editing at the human *CTNNB1* exon15. The exon sequence of *CTNNB1* gene is in gray shadow. The stop codon is indicated in red. The recognition site of sgCTNNB1 is in bold and underlined by a green line, The PAM is indicated in bold and the predicted cut site is indicated by a red triangle. The left homology arm is indicated with an orange line and right homology arm with a blue line. The precise length of homology arm is indicated under a thick arrow. Replaced sequences in left and right arms are separately indicated in purple and pink. Sequences in grey indicate genomic DNA outside of homology arms. The primers used for knockin pattern analysis by PCR are indicated by black arrows.

Supplementary Figure 3b: Precisely edited *CTNNB1* locus.

Primer CTNNB1-F1

Left homology arm

GTGGCCTGGCACTGAGTAATTACTTGGCTAAAGAAAGCTGGAGGTTGAAGAGGCTAGAAAGCGTTGTTTTCTGACAAGTTTGCTGCTGAACTTTGGATGCCCTAACCTCAG

308bp (HA300bp)

Exon15

TGTTAACGTCTATGTCTGCTTCTCTCCTCTCTCTTTTGCCTTCCTTCTTGCCTATTTTGTTGACACCCTGACTCTTCTAGATCCTAGCTATCGTTCTTTTCACTCTGGTGGATAT

140bp (HA150bp)

GGCCAGGATGCCTTGGGTATGGACCCCATGATGGAACAGAGATGGGTGGCCACCCACCCTGGTGCTGACTATCCAGTTGATGGGCTGCCAGATCTGGGGCATGCCCAGG

87bp (HA100bp)

***CTNNB1* with sense mutations** **GS Linker** **41bp (HA50bp)**

ACCTCATGGATGGGCTGGCCTCCtGGcGAtAGCAATCAGCTGGCCTGGTTTGATACTGACCTGGGTGGTGGTGGTTCTGGTGGTGGTGGTTCCGGCGGCGGCGGCTCTGGT

PAM **Destroyed recognition site of sgPRDM14**

mNeonGreen

GGTGGTGGATCCATGGTGAGCAAGGGCGAGGAGGATAACATGGCCTCTCTCCCAGCGACACATGAGTTACACATCTTTGGCTCCATCAACGGTGTGGACTTTGACATGGT

GGGTCAGGGCACCGGCAATCCAAATGATGGTTATGAGGAGTTAAACCTGAAGTCCACCAAGGGTGACCTCCAGTTCTCCCCCTGGATTCTGGTCCCTCATATCGGGTATG

GCTTCCATCAGTACCTGCCCTACCCTGACGGGATGTCGCCTTTCCAGGCCGCCATGGTAGATGGCTCCGGATACCAAGTCCATCGCACAATGCAGTTTGAAGATGGTGCC

TCCCTTACTGTTAACTACCGCTACACCTACGAGGGAAGCCACATCAAAGGAGAGGCCCAGGTGAAGGGGACTGGTTTCCCTGCTGACGGTCCTGTGATGACCAACTCGCT

GACCGCTGCGGACTGGTGCAGGTCGAAGAAGACTTACCCCAACGACAAAACCATCATCAGTACCTTTAAGTGGAGTTACACCACTGGAAATGGCAAGCGCTACCGGAGCA

CTGCGCGGACCACCTACACCTTTGCCAAGCCAATGGCGGCTAACTATCTGAAGAACCAGCCGATGTACGTGTTCCGTAAGACGGAGCTCAAGCACTCCAAGACCGAGCTC

Wpre

AACTTCAAGGAGTGGCAAAAGGCCTTTACCGATGTGATGGGCATGGACGAGCTGTACAAGTAAGTTTAAACGCGTCGACAATCAACCTCTGGATTACAAAATTTGTGAAAGA

TTGACTGGTATTCTTAACTATGTTGCTCCTTTTACGCTATGTGGATACGCTGCTTTAATGCCTTTGTATCATGCTATTGCTTCCCGTATGGCTTTCATTTTCTCCTCCTTGTATA

AATCCTGGTTGCTGTCTCTTTATGAGGAGTTGTGGCCCGTTGTCAGGCAACGTGGCGTGGTGTGCACTGTGTTTGCTGACGCAACCCCCACTGGTTGGGGCATTGCCACC

ACCTGTCAGCTCCTTTCCGGGACTTTCGCTTTCCCCCTCCCTATTGCCACGGCGGAACTCATCGCCGCCTGCCTTGCCCGCTGCTGGACAGGGGCTCGGCTGTTGGGCAC

TGACAATTCCGTGGTGTTGTCGGGGAAGCTGACGTCCTTTCCATGGCTGCTCGCCTGTGTTGCCACCTGGATTCTGCGCGGGACGTCCTTCTGCTACGTCCCTTCGGCCC

ployA

TCAATCCAGCGGACCTTCCTTCCCGCGGCCTGCTGCCGGCTCTGCGGCCTCTTCCGCGTCTTCGCCTTCGCCCTCGACGAGTCGGATCTCCCTTTGGGCGGATCCAGACA

TGATAAGATACATTGATGAGTTTGGACAAACCACAACTAGAATGCAGTGAAAAAAATGCTTTATTTGTGAAATTTGTGATGCTATTGCTTTATTTGTAACCATTATAAGCTGCA

ATAAACAAGTTAACAACAACAATTGCATTCATTTTATGTTTCAGGTTCAGGGGGAGGTGTGGGAGGTTTTTTAAAGCAAGTAAAACCTCTACAAATGTGGTATGGCTGATTAT

GATCCGGCTGCCTCGCGCGTTTCGGTGATGACGGTGAAAACCTCTGACACATGCAGCTCCCGGAGACGGTCACAGCTTGTCTGTAAGCGGATGCCGGGAGCAGACAAGC

Right homology arm

CCGTCAGGCGTCAGCGGGTGTTGGCGGGTGTCGGGGCGCAGCCATGAGTAAATCATCCTTTAGGTAAGAAGTTTTAAAAAGCCAGTTTGGGTAAAATACTTTTACTCTGCC

Stop Codon

51bp (HA50bp)

TACAGAACTTCAGAAAGACTTGGTTGGTAGGGTGGGAGTGGTTTAGGCTATTTGTAAATCTGCCACAAAAACAGGTATATACTTTGAAAGGAGATGTCTTGGAACATTGGAA

93bp (HA100bp) 145bp (HA150bp)

TGTTCTCAGATTTCTGGTTGTTATGTGATCATGTGTGGAAGTTATTAACTTTAATGTTTTTTGCCACAGCTTTTGCAACTTAATACTCAAATGAGTAACATTTGCTGTTTTAAAC

Primer CTNNB1-R1

ATTAATAGCAGCCTTTCTCTCTTTATACAGCTGTATTGTCTGAACTTGCATTGTGATTGGCCTGTAGAGTTGCTGAG

304bp (HA300bp)

Supplementary Figure 3b: Precisely edited *CTNNB1* locus. The exon of *CTNNB1* gene is in gray shadow. The stop codon is marked in red. Silent mutations are introduced to destroy the recognition site of sgCTNNB1, which is in bold and underlined by a green line. The left homology arm is indicated with an orange line and the right HA with a blue line. The precise length of homology arm is indicated under a thick arrow. The GS linker sequence is in yellow and mNeonGreen-Wpre-ployA sequence in green color. Sequences in grey indicate genomic DNA outside of homology arms. The primers used for knockin pattern analysis are indicated by black arrows.

Supplementary Figure 3c: Backbone sequence cut off from pD-mNeonGreen-sg (*CTNNB1*).

Partial recognition site of sgCTNNB1

Donor plasmid backbone sequence

ggtgacagcaatcagccaggtggcacttttcggggaaatgtgcgcggaacccctatttgtttatttttctaaatacattcaaatatgtatccgctcatgagacaataaccctgataaatgcttcaataatattgaa
aaaggaagagtatgagtattcaacatttccgtgtcgcccttattcccttttttgcggcattttgccttcctgtttttgctcacccagaaacgctggtgaaagtaaaagatgctgaagatcagttgggtgcacgagtgg
gttacatcgaactggatctcaacagcggtaagatccttgagagttttcgccccgaagaacgttttccaatgatgagcacttttaaagttctgctatgtggcgcggtattatcccgtattgacgccgggcaagagc
aactcggtcgccgcatacactattctcagaatgacttggttgagtactcaccagtcacagaaaagcatcttacggatggcatgacagtaagagaattatgcagtgctgccataaccatgagtgataacactg
cggccaacttacttctgacaacgatcggaggaccgaaggagctaaccgcttttttgcacaacatgggggatcatgtaactcgccttgatcgttgggaaccggagctgaatgaagccataccaaacgacga
gcgtgacaccacgatgcctgtagcaatggcaacaacgttgcgcaaactattaactggcgaactacttactctagcttcccggcaacaattaatagactggatggaggcggataaagttgcaggaccacttc
tgcgctcggcccttccggctggctggtttattgctgataaatctggagccggtgagcgtgggtctcgcggtatcattgcagcactggggccagatggtaagccctcccgtatcgtagttatctacacgacgggg
agtcaggcaactatggatgaacgaaatagacagatcgctgagataggtgcctcactgattaagcattggtaactgtcagaccaagtttactcatatatactttagattgatttaaaacttcatttttaatttaaaag
gatctaggtgaagatcctttttgataatctcatgaccaaaatcccttaacgtgagttttcgttccactgagcgtcagaccccgtagaaaagatcaaaggatcttcttgagatcctttttttctgcgcgtaatctgctgct
tgcaaacaaaaaaaccaccgctaccagcggtggtttgtttgccggatcaagagctaccaactctttttccgaaggtaactggcttcagcagagcgcagataccaaatactgtccttctagtgtagccgtagtta
ggccaccacttcaagaactctgtagcaccgcctacatacctcgctctgctaatcctgttaccagtggctgctgccagtggcgataagtcgtgtcttaccgggttggactcaagacgatagttaccggataaggc
gcagcggtcgggctgaacggggggttcgtgcacacagcccagcttggagcgaacgacctacaccgaactgagatacctacagcgtgagctatgagaaagcgccacgcttcccgaagggagaaagg
cggacaggtatccggtaagcggcagggtcggaacaggagagcgcacgagggagcttccagggggaaacgcctggtatctttatagtcctgtcgggtttcgccacctctgacttgagcgtcgatttttgtgat
gctcgtcaggggggcggagcctatggaaaaacgccagcaacgcggcctttttacggttcctggccttttgctggccttttgctcacatgttctttcctgcgttatcccctgattctgtggataaccgtattaccgcctttt
gagtgagctgataccgctcgccgcagccgaacgaccgagcgcagcgagtcagtgagcgaggaagcggaagagcgcccaatacgcaaaccgcctctccccgcgcgttggccgattcattaatgcagc
tggcacgacaggtttcccgactggaaagcgggcagtgagcgcaacgcaattaatgtgagttagctcactcattaggcaccccaggctttacactttatgcttccggctcgtatgttgtgtggaattgtgagcgg
ataacaatttcacaca**gctgattgctgtcacc**

Partial recognition site of sgCTNNB1

Supplementary Figure 3c: Backbone sequence cut off from pD-mNeonGreen-sg (*CTNNB1*). The sequence of donor plasmid backbone is in lower case and indicated by a red line. The residual partial sgCTNNB1 recognition sequence after linearization by CRISPR is in bold and indicated by green lines.

Supplementary Figure 3d: Knockin pattern analysis at the *CTNNB1* locus.

pD-mNeonGreen-sg-HA50-50bp (*CTNNB1*)

```
                Left homology arm                Replaced sequence in left arm              Replaced sequence in right arm                 Right homology arm
Wild type  TGCCAGATCTGGGGC::::::GGCTGCCTCCa--------------------------------------------------ggtgacagcaatcagctggcctggtttgatactgacctgTAAATCATCC::::GGTAAAATACTTTTA
                Left homology arm                CTNNB1 with sense mutations                 GS-mNeonGreen-Wpre-ployA                  Right homology arm
Expected   TGCCAGATCTGGGGC::::::GGCTGCCTCCtGGcGAtAGCAATCAGCTGGCCTGGTTTGATACTGACCTGGGTGGTGGTG::::::CAGCCATGAGTAAATCATCC::::GGTAAAATACTTTTA

Cl1-19     TGCCAGATCTGGGGC::::::GGCTGCCTCCtGGcGAtAGCAATCAGCTGGCCTGGTTTGATACTGACCTGGGTGGTGGTG::::::CAGCCATGAGTAAATCATCC::::GGTAAAATACTTTTA    HDR
Cl20-21    TGCCAGATCTGGGGC::::::GGCTGCCTCCtGGcGAtAGCAATCAGCTGGCCTGGTTTGATACTGACCTGGGTGGTGGTG::::::CAGCCATGAGtaaatcatcc::::::::::::::::::::ggtaaaatac
           ------cctggtttgatcatgacctgTAAATCATCC:::::GGTAAAATACTTTTA    3-NHEJ 71bp ins
Clone22    TGCCAGATCTGGGGC::::::GGCTGCCTCCtGGcGAtAGCAATCAGCTGGCCTGGTTTGATACTGACCTGGGTGGTGGTG:::::CAGCCATGAGtaaatcatcc::::::::::::::::::::ggt------
           -----gacagcaatcagctggcctggtttgatactgacctgTAAATCATCC:::::GGTAAAATACTTTTA    3-NHEJ 80bp ins
Clone23    TGCCAGATCTGGGGC::::::GGCTGCCTCCagtggagtattttacccaaactgg::::cctgggcatgccccagatcctccagggtgacagcaatcagctggcctggtttgatactgacctgTAAATCATCC:::::GGTAAAATACTTTTA    5,3-NHEJ mNeonGreen reverse insertion
Clone24    TGCCAGATCTGGGGC::::::GGCTGCCTCCaggacagcaatcagccaggtg:::::tcacacaggagctgattgctgtcaccggtgacagcaatcagctggcctggtttgatactgacctgTAAATCATCC:::::GGTAAAATACTTTTA    5,3-NHEJ Backbone forward insertion
Clone25    TGCCAGATCTGGGGC::::::GGCTGCCTCCagtggtgacagcaatcagccag::::tcacacaggagctgattgctgtcaccggtgacagcaatcagctggcctggtttgatactgacctgTAAATCATCC:::::GGTAAAATACTTTTA    5,3-NHEJ Backbone forward insertion
Clone26    TGCCAGATCTGGGGC::::::GGCTGCCT----cagcaatcagccaggtggcac::: ::tcacacaggagctgattgctgtcaccggtgacagcaatcagctggcctggtttgatactgacctgTAAATCATCC:::::GGTAAAATACTTTTA    5,3-NHEJ Backbone forward insertion
Cl27-28    TGCCAGATCTGGGGC::::::GGCTGCCTCCagggtgacagcaatcagctcctg::agtgccacctggctgattgctgtcaccggtgacagcaatcagctggcctggtttgatactgacctgTAAATCATCC:::::GGTAAAATACTTTTA    5,3-NHEJ Backbone reverse insertion
Clone29    TGCCAGATCTGGGGC::::::GGCTGCCT--------tcagctcctg::::agtgccacctggctgattgctgt----ggtgacagcaatcagctggcctggtttgatactgacctgTAAATCATCC:::::GGTAAAATACTTTTA    5,3-NHEJ Backbone reverse insertion
Clone30    TGCCAGATCTGGGGC:::::--------ggtgacagcaatcagctcctg:::ccccgaaaagtgccacctggctgatt--------gctggcctggtttgatactgacctgTAAATCATCC:::::GGTAAAATACTTTTA    5,3-NHEJ Backbone reverse insertion
```

pD-mNeonGreen-sg-HA100-100bp (*CTNNB1*)

```
                Left homology arm                Replaced sequence in left arm              Replaced sequence in right arm                 Right homology arm
Wild type  GAGATGGGTGGCCAC::::::GGCTGCCTCCa--------------------------------------------------ggtgacagcaatcagctggcctggtttgatactgacctgTAAATCATCC:::::TGGTTGGTAGGGTGG
                Left homology arm                CTNNB1 with sense mutations                 GS-mNeonGreen-Wpre-ployA                  Right homology arm
Expected   GAGATGGGTGGCCAC::::::GGCTGCCTCCtGGcGAtAGCAATCAGCTGGCCTGGTTTGATACTGACCTGGGTGGTGGTG::::CAGCCATGAGTAAATCATCC::::TGGTTGGTAGGGTGG

Cl1-16     GAGATGGGTGGCCAC::::::GGCTGCCTCCtGGcGAtAGCAATCAGCTGGCCTGGTTTGATACTGACCTGGGTGGTGGTG::::CAGCCATGAGTAAATCATCC:::::TGGTTGGTAGGGTGG    HDR
Clone17    GAGATGGGTGGCCAC::::::GGCTGCCTCCtGGcGAtAGCAATCAGCTGGCCTGGTTTGATACTGACCTGGGTGGTGGTG::::CAGCCATGAGtaaatcatcc::::::::::::::::::::::tggttggt--
           ------tggcctggtttgatactgacctgTAAATCATCC::::TGGTTGGTAGGGTGG    3-NHEJ 114bp ins
Clone18    GAGATGGGTGGCCAC::::::GGCTGCCTCCtGGcGAtAGCAATCAGCTGGCCTGGTTTGATACTGACCTGGGTGGTGGTG::::------
           ------caatcagctggcctggtttgatactgacctgTAAATCATCC::::TGGTTGGTAGGGTGG    3-NHEJ 493bp del; 31bp ins
Clone19    GAGATGGGTGGCCAC::::: --------tggagggtggccac::::ggctgcctcctggcgatagcaatcagctggcctggtttgatactgacctgggtggtggtg::cagccatgagTAAATCATCC:::::TGGTTGGTAGGGTGG    5-NHEJ 41bp del; 131bp ins
Clone20    GAGATGGGTGGCCAC::::::GGCTGCCTCCatggagggtggccac::::ggctgcctcctggcgatagcaatcagctggcctggtttgatactgacctgggtggtggtg::cagccatgagtaaatcatcc::::::::::::::::::::::tggttggtagcctccag
           ggtgacagcaatcagctggcctggtttgatactgacctgTAAATCATCC:::::TGGTTGGTAGGGTGG    5-NHEJ 91bp ins; 3-NHEJ 139bp ins
Clone21    GAGATGGGTGGCCAC::::::GGCTGCCTCCaggtgacagcaatcagccaggtg:::::cacaggagctgattgctgtcaccggtgacagcaatcagctggcctggtttgatactgacctgTAAATCATCC:::::TGGTTGGTAGGGTGG    5,3-NHEJ Backbone forward insertion
Cl22-23    GAGATGGGTGGCCAC::::::GGCTGCCTCCagggtgacagcaatcagccaggtg:::cacaggagctgattgctgtcaccggtgacagcaatcagctggcctggtttgatactgacctgTAAATCATCC:::::TGGTTGGTAGGGTGG    5,3-NHEJ Backbone forward insertion
Clone24    GAGATGGGTGGCCAC::::::GGCTGCCTCC-ggtgacagcaatcagccaggtg:::::cacaggagctgattgctgtcaccggtgacagcaatcagctggcctggtttgatactgacctgTAAATCATCC:::::TGGTTGGTAGGGTGG    5,3-NHEJ Backbone forward insertion
Cl25-26    GAGATGGGTGGCCAC::::::GGCTGCCTCC-------agcaatcagccaggtg:::: :::cacaggagctgattgctgtcaccggtgacagcaatcagctggcctggtttgatactgacctgTAAATCATCC:::::TGGTTGGTAGGGTGG    5,3-NHEJ Backbone forward insertion
Clone27    GAGATGGGTGGCCAC::::::GGCTGCCT------gacagcaatcagccaggtg:::::cacaggagctgattgctgtca--------tcagctggcctggtttgatactgacctgTAAATCATCC:::::TGGTTGGTAGGGTGG    5,3-NHEJ Backbone forward insertion
Clone28    GAGATGGGTGGCCAC::::::GGCTGCCTCCa---gacagcaatcagccaggtg:::::cacaggagctgattgctg--------gcctggtttgatactgacctgTAAATCATCC:::::TGGTTGGTAGGGTGG    5,3-NHEJ Backbone forward insertion
Clone29    GAGATGGGTGGCCAC::::::GGCTGCCTCCag----------cagccaggtg:::::cacaggagctgattgctgtcaccagctgctgatt
           -gtgacagcaatcagctggcctggtttgatactgacctgTAAATCATCC:::::TGGTTGGTAGGGTGG    5,3-NHEJ Backbone forward insertion
Clone30    GAGATGGGTGGCCAC::::::GGCTGCCTCCagggtgacagcaatcagctcctgtgt::::cacctggctgattgctgtcaccggtgacagcaatcagctggcctggtttgatactgacctgTAAATCATCC:::::TGGTTGGTAGGGTGG    5,3-NHEJ Backbone reverse insertion
```

pD-mNeonGreen-sg-HA150-150bp (*CTNNB1*)

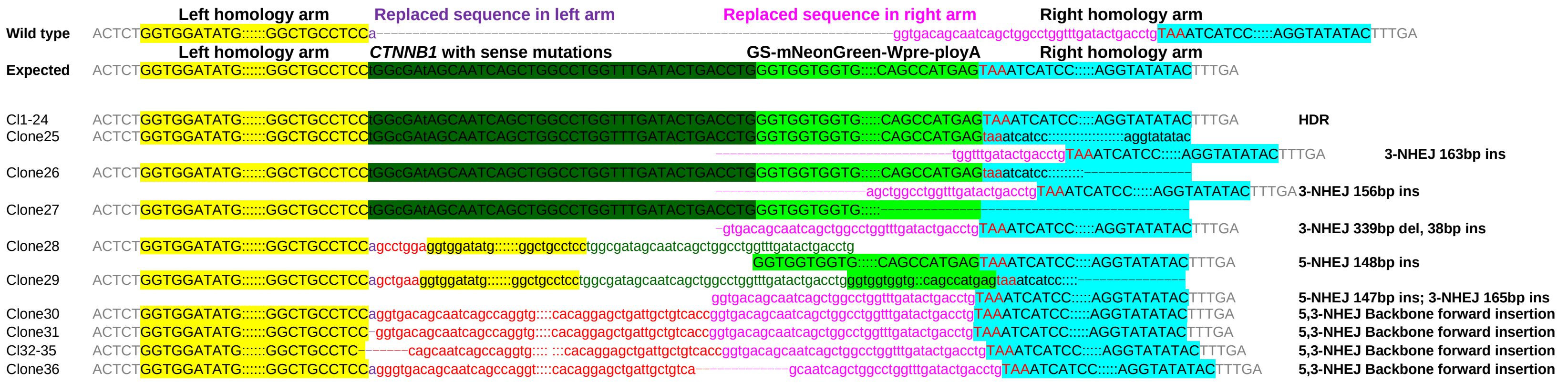

pD-mNeonGreen-sg-HA300-300bp (*CTNNB1*)

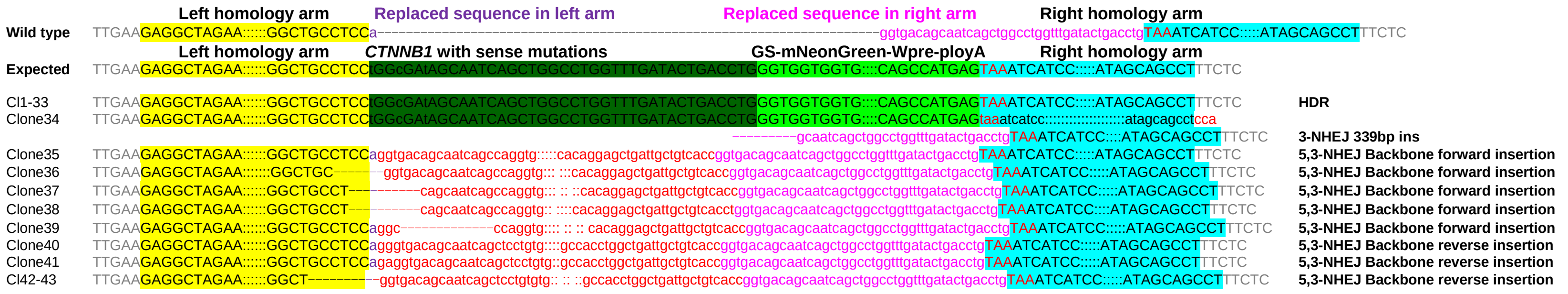

Supplementary Figure 3d: Knockin pattern analysis at the *CTNNB1* locus. The stop codon is indicated in red. The left homology arm is highlighted in yellow shadow and right homology arm in blue shadow. Replaced sequences at upstream and

downstream of DSB are separately indicated in purple and pink lowercase. The inserted sequence is in red lowercase. Sequences in grey indicate genomic DNA outside of homology arms.

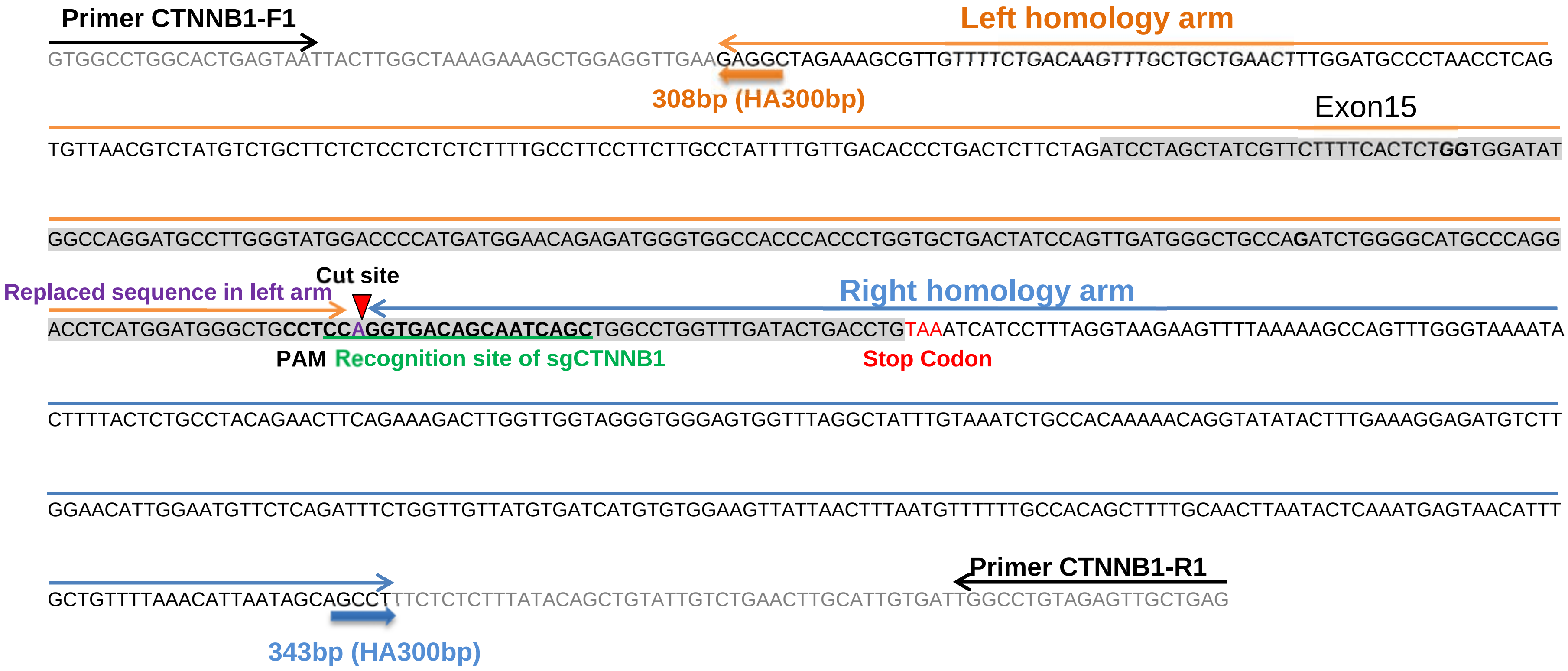

Supplementary Figure 4a: Improved design of genome editing at human *CTNNB1* exon15 (pD-mNeonGreen-sg-HA300-300bp-RR1-0bp). The exon sequence of *CTNNB1* is in gray shadow. The stop codon is indicated in red. The recognition site of sgCTNNB1 is in bold and underlined by a green line. The PAM is indicated in bold and the expected cut site is indicated by a green triangle. The left homology arm is indicated with an orange line and the right arm with a blue line. The precise length of homology arm is indicated under a thick arrow. Replaced sequence at upstream of DSB is indicated in purple. Sequences in grey indicate genomic DNA outside of homology arms. The primers used for knockin pattern analysis are indicated by black arrows.

Supplementary Figure 4b: Precisely edited *CTNNB1* locus with pD-mNeonGreen-sg-HA300-300bp-RR1-0bp

Primer CTNNB1-F1 → **Left homology arm**

GTGGCCTGGCACTGAGTAATTACTTGGCTAAAGAAAGCTGGAGGTTGAAGAGGCTAGAAAGCGTTGTTTTCTGACAAGTTTGCTGCTGAACTTTGGATGCCCTAACCTCAG

308bp (HA300bp) **Exon15**

TGTTAACGTCTATGTCTGCTTCTCTCCTCTCTCTTTTGCCTTCCTTCTTGCCTATTTTGTTGACACCCTGACTCTTCTAGATCCTAGCTATCGTTCTTTTCACTCTGGTGGATAT

GGCCAGGATGCCTTGGGTATGGACCCCATGATGGAACAGAGATGGGTGGCCACCCACCCTGGTGCTGACTATCCAGTTGATGGGCTGCCAGATCTGGGGCATGCCCAGG

***CTNNB1* with sense mutations** **GS Linker**

ACCTCATGGATGGGCTG**CCTCC**tGGcGAtAGCAATCAGCTGGCCTGGTTTGATACTGACCTGGGTGGTGGTGGTTCTGGTGGTGGTGGTTCCGGCGGCGGCGGCTCTGGT

PAM **Destroyed recognition site of sgCTNNB1**

mNeonGreen

GGTGGTGGATCCATGGTGAGCAAGGGCGAGGAGGATAACATGGCCTCTCTCCCAGCGACACATGAGTTACACATCTTTGGCTCCATCAACGGTGTGGACTTTGACATGGT

GGGTCAGGGCACCGGCAATCCAAATGATGGTTATGAGGAGTTAAACCTGAAGTCCACCAAGGGTGACCTCCAGTTCTCCCCCTGGATTCTGGTCCCTCATATCGGGTATG

GCTTCCATCAGTACCTGCCCTACCCTGACGGGATGTCGCCTTTCCAGGCCGCCATGGTAGATGGCTCCGGATACCAAGTCCATCGCACAATGCAGTTTGAAGATGGTGCC

TCCCTTACTGTTAACTACCGCTACACCTACGAGGGAAGCCACATCAAAGGAGAGGCCCAGGTGAAGGGGACTGGTTTCCCTGCTGACGGTCCTGTGATGACCAACTCGCT

GACCGCTGCGGACTGGTGCAGGTCGAAGAAGACTTACCCCAACGACAAAACCATCATCAGTACCTTTAAGTGGAGTTACACCACTGGAAATGGCAAGCGCTACCGGAGCA

CTGCGCGGACCACCTACACCTTTGCCAAGCCAATGGCGGCTAACTATCTGAAGAACCAGCCGATGTACGTGTTCCGTAAGACGGAGCTCAAGCACTCCAAGACCGAGCTC

Wpre

AACTTCAAGGAGTGGCAAAAGGCCTTTACCGATGTGATGGGCATGGACGAGCTGTACAAGTAAGTTTAAACGCGTCGACAATCAACCTCTGGATTACAAAATTTGTGAAAGA

TTGACTGGTATTCTTAACTATGTTGCTCCTTTTACGCTATGTGGATACGCTGCTTTAATGCCTTTGTATCATGCTATTGCTTCCCGTATGGCTTTCATTTTCTCCTCCTTGTATA

AATCCTGGTTGCTGTCTCTTTATGAGGAGTTGTGGCCCGTTGTCAGGCAACGTGGCGTGGTGTGCACTGTGTTTGCTGACGCAACCCCCACTGGTTGGGGCATTGCCACC

ACCTGTCAGCTCCTTTCCGGGACTTTCGCTTTCCCCCTCCCTATTGCCACGGCGGAACTCATCGCCGCCTGCCTTGCCCGCTGCTGGACAGGGGCTCGGCTGTTGGGCAC

TGACAATTCCGTGGTGTTGTCGGGGAAGCTGACGTCCTTTCCATGGCTGCTCGCCTGTGTTGCCACCTGGATTCTGCGCGGGACGTCCTTCTGCTACGTCCCTTCGGCCC

ployA

TCAATCCAGCGGACCTTCCTTCCCGCGGCCTGCTGCCGGCTCTGCGGCCTCTTCCGCGTCTTCGCCTTCGCCCTCGACGAGTCGGATCTCCCTTTGGGCGGATCCAGACA

TGATAAGATACATTGATGAGTTTGGACAAACCACAACTAGAATGCAGTGAAAAAAATGCTTTATTTGTGAAATTTGTGATGCTATTGCTTTATTTGTAACCATTATAAGCTGCA

ATAAACAAGTTAACAACAACAATTGCATTCATTTTATGTTTCAGGTTCAGGGGGAGGTGTGGGAGGTTTTTTAAAGCAAGTAAAACCTCTACAAATGTGGTATGGCTGATTAT

GATCCGGCTGCCTCGCGCGTTTCGGTGATGACGGTGAAAACCTCTGACACATGCAGCTCCCGGAGACGGTCACAGCTTGTCTGTAAGCGGATGCCGGGAGCAGACAAGC

Right homology arm

CCGTCAGGCGTCAGCGGGTGTTGGCGGGTGTCGGGGCGCAGCCATGAGGGTGACAGCAATCAGCTGGCCTGGTTTGATACTGACCTGTAAATCATCCTTTAGGTAAGAA

Stop Codon

GTTTTAAAAAGCCAGTTTGGGTAAAATACTTTTACTCTGCCTACAGAACTTCAGAAAGACTTGGTTGGTAGGGTGGGAGTGGTTTAGGCTATTTGTAAATCTGCCACAAAAAC

AGGTATATACTTTGAAAGGAGATGTCTTGGAACATTGGAATGTTCTCAGATTTCTGGTTGTTATGTGATCATGTGTGGAAGTTATTAACTTTAATGTTTTTTGCCACAGCTTTT

Primer CTNNB1-R1

GCAACTTAATACTCAAATGAGTAACATTTGCTGTTTTAAACATTAATAGCAGCCTTTCTCTCTTTATACAGCTGTATTGTCTGAACTTGCATTGTGATTGGCCTGTAGAGTTGC

343bp (HA300bp)

TGAG

Supplementary Figure 4b: Precisely edited *CTNNB1* locus with pD-mNeonGreen-sg-HA300-300bp-RR1-0bp. The exon sequence of *CTNNB1* is in gray shadow. The stop codon is indicated in red. Silent mutations (indicated in lowercase) were introduced to destroy the recognition site of sgCTNNB1, which is in bold and underlined by a green line. The PAM is indicated in bold. The left homology arm is indicated with an orange line and right homology arm with a blue line. The precise lengths of homology arm are indicated under thick arrows. The GS linker sequence is in yellow and mNeonGreen-Wpre-ployA sequence in green color. Sequences in grey indicate genomic DNA outside of homology arms. The primers used for knockin pattern analysis are indicated by black arrows.

pD-mNeonGreen-sg-HA300-300bp-RR1-0bp (*CTNNB1*)

Left homology arm Replaced sequence in left arm Right homology arm

Wild type TTGAAGAGGCTAGAA::::::GGCTGCCTCCa---
GGTGACAGCAATCAGCTGGCCTGGTTTGATACTGACCTGTAAATCATCC:::::ATAGCAGCCTTTCTC

Left homology arm *CTNNB1* with sense mutations GS-mNeonGreen-Wpre-ployA Right homology arm

Expected TTGAAGAGGCTAGAA::::::GGCTGCCTCCtGGcGAtAGCAATCAGCTGGCCTGGTTTGATACTGACCTGGGTGGTGGTG:::::CAGCCATGAGGGTGACAGCAATCAGCTGGCCTGGTTTGATACTGACCTGTAAATCATCC:::::ATAGCAGCCTTTCTC

Cl1-37 TTGAAGAGGCTAGAA::::::GGCTGCCTCCtGGcGAtAGCAATCAGCTGGCCTGGTTTGATACTGACCTGGGTGGTGGTG:::::CAGCCATGAGGGTGACAGCAATCAGCTGGCCTGGTTTGATACTGACCTGTAAATCATCC:::::ATAGCAGCCTTTCTC
HDR

Clone38 TTGAAGAGGCTAGAA::::::GGCTGCCTCCagtggaggctagaa:::::ggctgcctcc
tGGcGAtAGCAATCAGCTGGCCTGGTTTGATACTGACCTGGGTGGTGGTG:::::CAGCCATGAGGGTGACAGCAATCAGCTGGCCTGGTTTGATACTGACCTGTAAATCATCC:::::ATAGCAGCCTTTCTC
3-NHEJ 312bp ins

Clone39 TTGAAGAGGCTAGAA::::::----------------tggaggctagaa::::::ggctgcctcc
tGGcGAtAGCAATCAGCTGGCCTGGTTTGATACTGACCTGGGTGGTGGTG:::::CAGCCATGAGGGTGACAGCAATCAGCTGGCCTGGTTTGATACTGACCTGTAAATCATCC:::::ATAGCAGCCTTTCTC
3-NHEJ 13bp del, 310bp ins

Clone40 TTGAAGAGGCTAGAA::::::-------------------------------: : : : : :----------
----
:::CAGCCATGAGGGTGACAGCAATCAGCTGGCCTGGTTTGATACTGACCTGTAAATCATCC:::::ATAGCAGCCTTTCTC
3-NHEJ 176bp del

Clone41 TTGAAGAGGCTAGAA::::::-----------------------agcaatcagccaggtggcacttttcggggaaatgtgcgc:::::::::: ::::::::: :::::::: :::::::::::
:::::::acacaggagctgattgctgtcaccGGTGACAGCAATCAGCTGGCCTGGTTTGATACTGACCTGTAAATCATCC:::::ATAGCAGCCTTTCTC
5,3-NHEJ Backbone reverse insertion

Clone42 TTGAAGAGGCTAGAA::::::GGCTGCCTCCag---
:::acacaggagctgattgctgtcaccGGTGACAGCAATCAGCTGGCCTGGTTTGATACTGACCTGTAAATCATCC:::::ATAGCAGCCTTTCTC
5,3-NHEJ Backbone reverse insertion

Supplementary Figure 4c: Knockin pattern analysis at the *CTNNB1* locus edited by pD-mNeonGreen-sg-HA300-300bp-RR1-0bp (*CTNNB1*). The stop codon is indicated in red. The left homology arm is indicated in yellow shadow and right homology arm in blue shadow. Replaced sequence in the left arm is indicated in purple lowercase. The inserted sequence is in red lowercase. Sequences in grey indicate genomic DNA outside of homology arms.

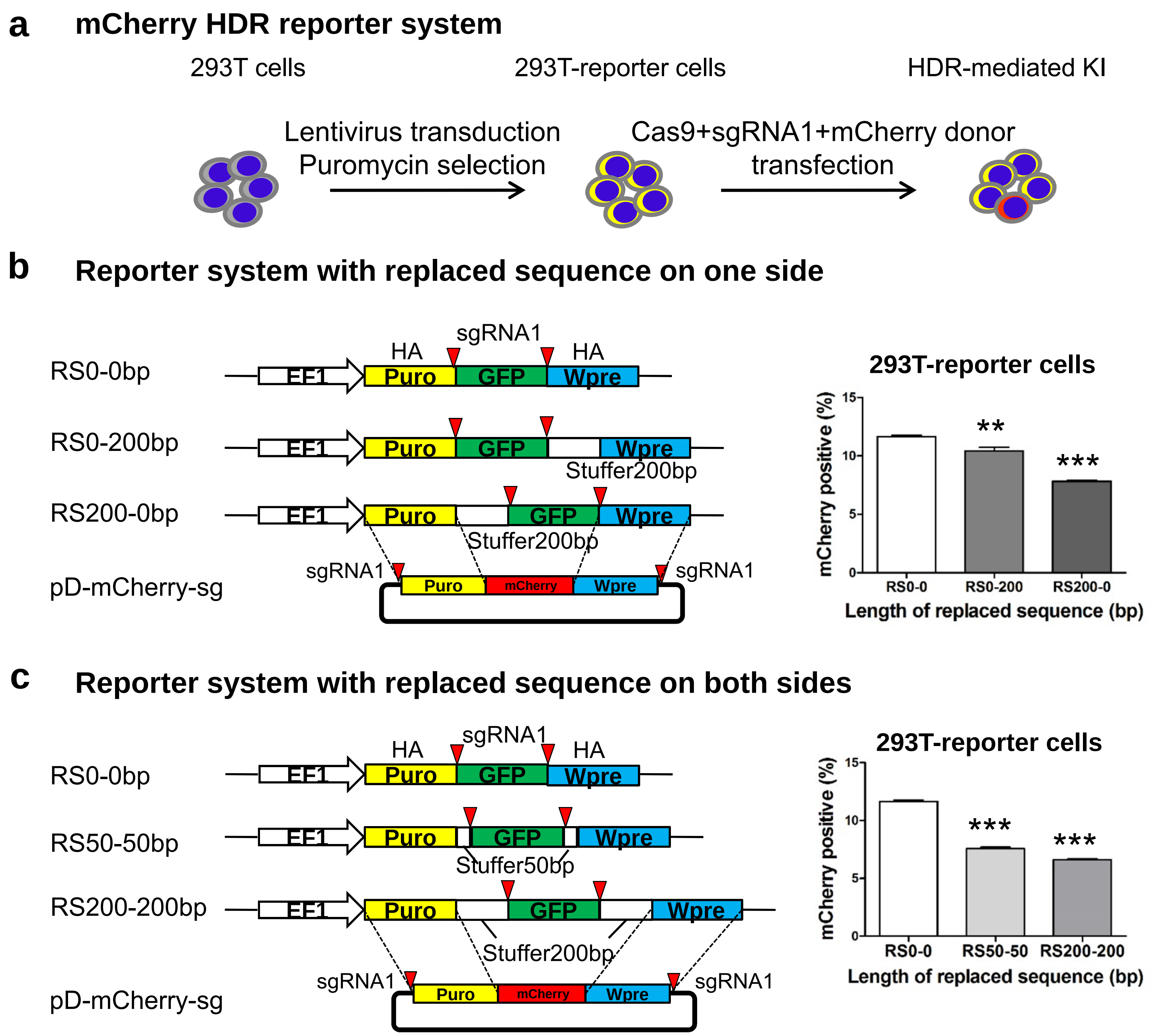
a
mCherry HDR reporter system
293T cells
293T-reporter cells
HDR-mediated KI
Lentivirus transduction
Puromycin selection
Cas9+sgRNA1+mCherry donor
transfection
b
Reporter system with replaced sequence on one side
RS0-0bp
RS0-200bp
RS200-0bp
pD-mCherry-sg
HA
sgRNA1
HA
EF1
Puro
GFP
Wpre
Stuffer200bp
Stuffer200bp
sgRNA1
sgRNA1
mCherry
293T-reporter cells
mCherry positive (%)
15
10
5
0
**
***
RS0-0
RS0-200
RS200-0
Length of replaced sequence (bp)
c
Reporter system with replaced sequence on both sides
RS0-0bp
RS50-50bp
RS200-200bp
pD-mCherry-sg
HA
sgRNA1
HA
EF1
Puro
GFP
Wpre
Stuffer50bp
Stuffer200bp
sgRNA1
sgRNA1
mCherry
293T-reporter cells
mCherry positive (%)
15
10
5
0
***
***
RS0-0
RS50-50
RS200-200
Length of replaced sequence (bp)

Supplementary Figure 5: The effects of replaced sequence length adjacent to DSB site (sgRNA1 cleavage site) on HDR efficiency. (**a**) Schematic outline of the HDR reporter systems. 293T cells transduced with lentiviral vectors harboring different lengths of replaced sequences in (**b**) and (**c**) were used as reporter cell lines. (**b-c**) The HDR-mediated knockin efficiency was determined by FACS at day 7 after co-transfection with Cas9/sgRNA1 and the donor plasmid pD-mCherry-sg-HA300-300bp. Significance was calculated using Student's paired t-test: **$P \leq 0.01$; ***$P \leq 0.001$.

Supplementary Figure 6a: Design of genome editing at the *PRDM14* locus

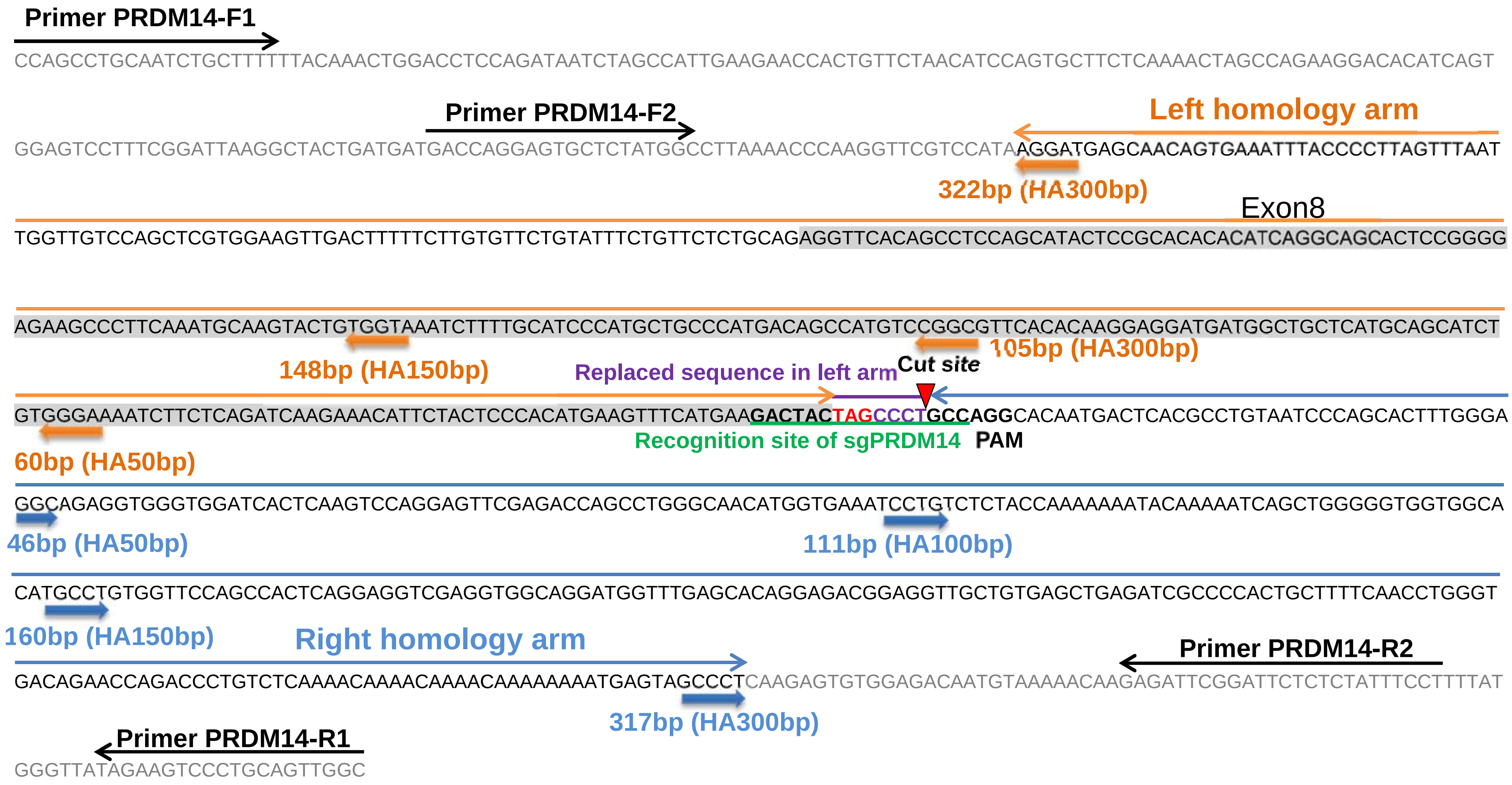

Supplementary Figure 6a: Design of genome editing at the human *PRDM14* exon8. The exon8 of *PRDM14* is indicated in gray shadow. The stop codon is indicated in red. The recognition site of sgPRDM14 is in bold and underlined by a green line. The PAM is indicated in bold and the cut site is indicated by a red triangle. The left homology arm is indicated with an orange line and right homology arm with a blue line. The precise length of homology arm is indicated under a thick arrow. Replaced sequence upstream of DSB is indicated in purple. Sequences in grey indicate genomic DNA outside of homology arms. The two pairs of primers used for knockin pattern analysis by PCR are indicated by black arrows.

Supplementary Figure 6b: Precisely edited *PRDM14* locus.

Primer PRDM14-F1

CCAGCCTGCAATCTGCTTTTTTACAAACTGGACCTCCAGATAATCTAGCCATTGAAGAACCACTGTTCTAACATCCAGTGCTTCTCAAAACTAGCCAGAAGGACACATCAGT

Primer PRDM14-F2 **Left homology arm**

GGAGTCCTTTCGGATTAAGGCTACTGATGATGACCAGGAGTGCTCTATGGCCTTAAAACCCAAGGTTCGTCCATAAGGATGAGCAACAGTGAAATTTACCCCTTAGTTTAAT

322bp (HA300bp) Exon8

TGGTTGTCCAGCTCGTGGAAGTTGACTTTTTCTTGTGTTCTGTATTTCTGTTCTCTGCAGAGGTTCACAGCCTCCAGCATACTCCGCACACACATCAGGCAGCACTCCGGGG

AGAAGCCCTTCAAATGCAAGTACTGTGGTAAATCTTTTGCATCCCATGCTGCCCATGACAGCCATGTCCGGCGTTCACACAAGGAGGATGATGGCTGCTCATGCAGCATCT

148bp (HA150bp) **105bp (HA100bp)** **2A**

GTGGGAAAATCTTCTCAGATCAAGAAACATTCTACTCCCACATGAAGTTTCATGAA**GACTAC**CAGTGTACTAATTATGCTCTCTTGAAATTGGCTGGAGATGTTGAGAGCAAC

Destroyed recognition site of sgPRDM14

60bp (HA50bp) **GFP**

CCAGGTCCCATGGTGAGCAAGGGCGAGGAGCTGTTCACCGGGGTGGTGCCCATCCTGGTCGAGCTGGACGGCGACGTAAACGGCCACAAGTTCAGCGTGTCCGGCGAG

GGCGAGGGCGATGCCACCTACGGCAAGCTGACCCTGAAGTTCATCTGCACCACCGGCAAGCTGCCCGTGCCCTGGCCCACCCTCGTGACCACCCTGACCTACGGCGTG

CAGTGCTTCAGCCGCTACCCCGACCACATGAAGCAGCACGACTTCTTCAAGTCCGCCATGCCCGAAGGCTACGTCCAGGAGCGCACCATCTTCTTCAAGGACGACGGCAA

CTACAAGACCCGCGCCGAGGTGAAGTTCGAGGGCGACACCCTGGTGAACCGCATCGAGCTGAAGGGCATCGACTTCAAGGAGGACGGCAACATCCTGGGGCACAAGCT

GGAGTACAACTACAACAGCCACAACGTCTATATCATGGCCGACAAGCAGAAGAACGGCATCAAGGTGAACTTCAAGATCCGCCACAACATCGAGGACGGCAGCGTGCAGC

TCGCCGACCACTACCAGCAGAACACCCCCATCGGCGACGGCCCCGTGCTGCTGCCCGACAACCACTACCTGAGCACCCAGTCCGCCCTGAGCAAAGACCCCAACGAGAA

Wpre

GCGCGATCACATGGTCCTGCTGGAGTTCGTGACCGCCGCCGGGATCACTCTCGGCATGGACGAGCTGTACAAGTAAGTTTAAACGCGTCGACAATCAACCTCTGGATTAC

AAAATTTGTGAAAGATTGACTGGTATTCTTAACTATGTTGCTCCTTTTACGCTATGTGGATACGCTGCTTTAATGCCTTTGTATCATGCTATTGCTTCCCGTATGGCTTTCATTT

TCTCCTCCTTGTATAAATCCTGGTTGCTGTCTCTTTATGAGGAGTTGTGGCCCGTTGTCAGGCAACGTGGCGTGGTGTGCACTGTGTTTGCTGACGCAACCCCCACTGGTT

GGGGCATTGCCACCACCTGTCAGCTCCTTTCCGGGACTTTCGCTTTCCCCCTCCCTATTGCCACGGCGGAACTCATCGCCGCCTGCCTTGCCCGCTGCTGGACAGGGGCT

CGGCTGTTGGGCACTGACAATTCCGTGGTGTTGTCGGGGAAGCTGACGTCCTTTCCATGGCTGCTCGCCTGTGTTGCCACCTGGATTCTGCGCGGGACGTCCTTCTGCTA

CGTCCCTTCGGCCCTCAATCCAGCGGACCTTCCTTCCCGCGGCCTGCTGCCGGCTCTGCGGCCTCTTCCGCGTCTTCGCCTTCGCCCTCGACGAGTCGGATCTCCCTTTG

PolyA

GGCGGATCCAGACATGATAAGATACATTGATGAGTTTGGACAAACCACAACTAGAATGCAGTGAAAAAAATGCTTTATTTGTGAAATTTGTGATGCTATTGCTTTATTTGTAAC

CATTATAAGCTGCAATAAACAAGTTAACAACAACAATTGCATTCATTTTATGTTTCAGGTTCAGGGGGAGGTGTGGGAGGTTTTTTAAAGCAAGTAAAACCTCTACAAATGTG

GTATGGCTGATTATGATCCGGCTGCCTCGCGCGTTTCGGTGATGACGGTGAAAACCTCTGACACATGCAGCTCCCGGAGACGGTCACAGCTTGTCTGTAAGCGGATGCCG

GGAGCAGACAAGCCCGTCAGGGCGCGTCAGCGGGTGTTGGCGGGTGTCGGGGCGCAGCCATGAG**GCCAGG**CACAATGACTCACGCCTGTAATCCCAGCACTTTGGGAG

PAM

GCAGAGGTGGGTGGATCACTCAAGTCCAGGAGTTCGAGACCAGCCTGGGCAACATGGTGAAATCCTGTCTCTACCAAAAAAATACAAAAATCAGCTGGGGGTGGTGGCAC

46bp (HA50bp) 111bp (HA100bp)

ATGCCTGTGGTTCCAGCCACTCAGGAGGTCGAGGTGGCAGGATGGTTTGAGCACAGGAGACGGAGGTTGCTGTGAGCTGAGATCGCCCCACTGCTTTTCAACCTGGGTG

160bp (HA150bp) Right homology arm Primer PRDM14-R2

ACAGAACCAGACCCTGTCTCAAAACAAAACAAAACAAAACAAAACAAAAAAAATGAGTAGCCCTCAAGAGTGTGGAGACAATGTAAAAACAAGAGATTCGGATTCTCTCTATT

Primer PRDM14-R1 317bp (HA300bp)

TCCTTTTATGGGTTATAGAAGTCCCTGCAGTTGGC

Supplementary Figure 6b: Precisely edited *PRDM14* locus. The exon8 of *PRDM14* is indicated in gray shadow. The stop codon is indicated in red. The destroyed recognition site of sgPRDM14 is in bold and underlined by a green line, and PAM is indicated in bold. The left homology arm is indicated with an orange line and right homology arm with a blue line. The precise lengths of homology arm are indicated with thick arrows. The ribosome-skipping sequence 2A is in yellow and GFP-Wpre-ployA sequence in green color. Sequences in grey indicate genomic DNA outside of homology arms. The two pairs of primers used for knockin pattern analysis by PCR are indicated by black arrows.

Supplementary Figure 6c: Backbone sequence cut off form pD-GFP-sg (*PRDM14*)

Partial recognition site of sgPRDM14

Donor plasmid backbone sequence

agggctagtagtcttccaggtggcacttttcggggaaatgtgcgcggaacccctatttgtttatttttctaaatacattcaaatatgtatccgctcatgagacaataaccctgataaatgcttcaataatattgaaa
aaggaagagtatgagtattcaacatttccgtgtcgcccttattcccttttttgcggcattttgccttcctgtttttgctcacccagaaacgctggtgaaagtaaaagatgctgaagatcagttgggtgcacgagtggg
ttacatcgaactggatctcaacagcggtaagatccttgagagttttcgccccgaagaacgttttccaatgatgagcacttttaaagttctgctatgtggcgcggtattatcccgtattgacgccgggcaagagca
actcggtcgccgcatacactattctcagaatgacttggttgagtactcaccagtcacagaaaagcatcttacggatggcatgacagtaagagaattatgcagtgctgccataaccatgagtgataacactgc
ggccaacttacttctgacaacgatcggaggaccgaaggagctaaccgcttttttgcacaacatgggggatcatgtaactcgccttgatcgttgggaaccggagctgaatgaagccataccaaacgacgag
cgtgacaccacgatgcctgtagcaatggcaacaacgttgcgcaaactattaactggcgaactacttactctagcttcccggcaacaattaatagactggatggaggcggataaagttgcaggaccacttct
gcgctcggcccttccggctggctggtttattgctgataaatctggagccggtgagcgtgggtctcgcggtatcattgcagcactggggccagatggtaagccctcccgtatcgtagttatctacacgacgggga
gtcaggcaactatggatgaacgaaatagacagatcgctgagataggtgcctcactgattaagcattggtaactgtcagaccaagtttactcatatatactttagattgatttaaaacttcatttttaatttaaaagg
atctaggtgaagatcctttttgataatctcatgaccaaaatcccttaacgtgagttttcgttccactgagcgtcagaccccgtagaaaagatcaaaggatcttcttgagatcctttttttctgcgcgtaatctgctgctt
gcaaacaaaaaaaccaccgctaccagcggtggtttgtttgccggatcaagagctaccaactctttttccgaaggtaactggcttcagcagagcgcagataccaaatactgtccttctagtgtagccgtagtta
ggccaccacttcaagaactctgtagcaccgcctacatacctcgctctgctaatcctgttaccagtggctgctgccagtggcgataagtcgtgtcttaccgggttggactcaagacgatagttaccggataaggc
gcagcggtcgggctgaacggggggttcgtgcacacagcccagcttggagcgaacgacctacaccgaactgagatacctacagcgtgagctatgagaaagcgccacgcttcccgaagggagaaagg
cggacaggtatccggtaagcggcagggtcggaacaggagagcgcacgagggagcttccagggggaaacgcctggtatctttatagtcctgtcgggtttcgccacctctgacttgagcgtcgatttttgtgat
gctcgtcaggggggcggagcctatggaaaaacgccagcaacgcggcctttttacggttcctggccttttgctggccttttgctcacatgttctttcctgcgttatcccctgattctgtggataaccgtattaccgcttt
gagtgagctgataccgctcgccgcagccgaacgaccgagcgcagcgagtcagtgagcgaggaagcggaagagcgcccaatacgcaaaccgcctctccccgcgcgttggccgattcattaatgcagc
tggcacgacaggtttcccgactggaaagcgggcagtgagcgcaacgcaattaatgtgagttagctcactcattaggcaccccaggctttacactttatgcttccggctcgtatgttgtgtggaattgtgagcgg
ataacaatttcacaca**gaagactactagccct**

Partial recognition site of sgPRDM14

Supplementary Figure 6c: Backbone sequence cut off from pD-mNeonGreen-sg (*PRDM14*). The sequence of donor plasmid backbone is in lowercase and indicated by a red line. The residual partial sgPRDM14 recognition sequence after plasmid linearization by CRISPR is in bold and indicated by green lines.

Supplementary Figure 6d: Knockin pattern analysis at the *PRDM14* locus.

pD-GFP-sg-HA50-50bp (*PRDM14*)

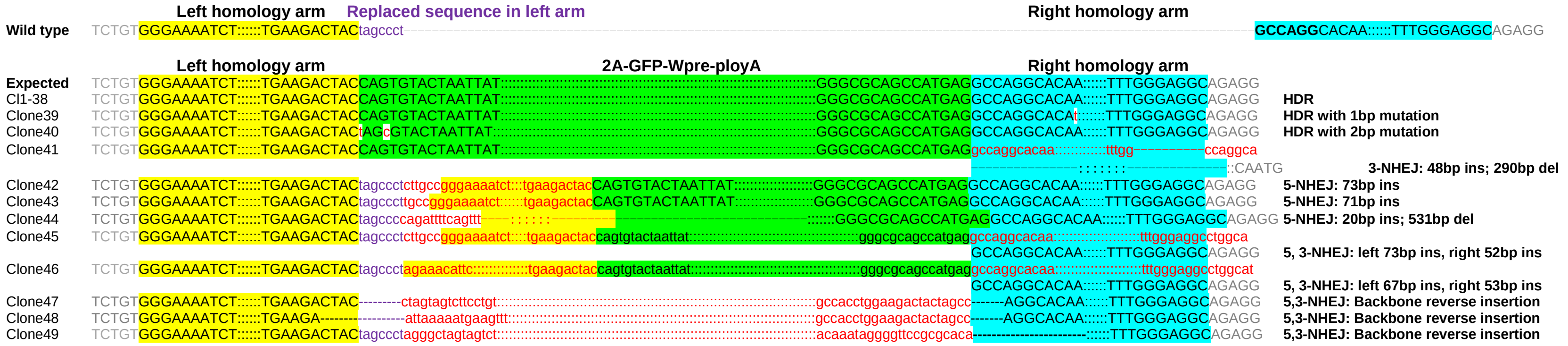

pD-GFP-sg-HA100-100bp (*PRDM14*)

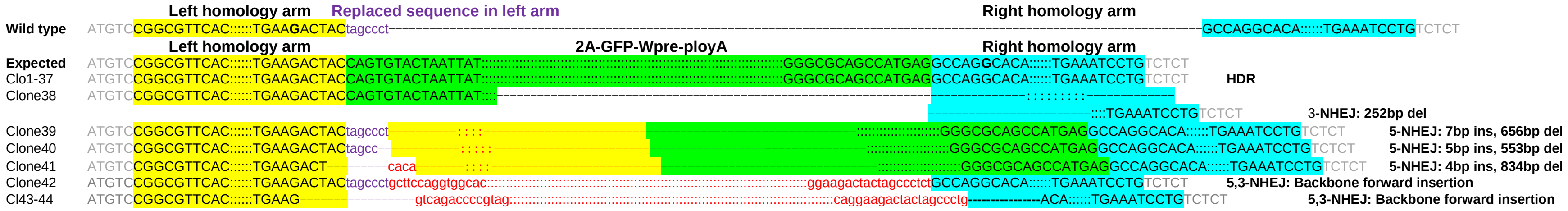

pD-GFP-sg-HA150-150bp (*PRDM14*)

	Left homology arm / 2A-GFP-Wpre-ployA / Right homology arm	
**Wild type**	TACTG TGGTAAATCT::::::TGAAGACTAC tagccct-- GCCAGGCACA::::::GCACATGCCT GTGGT	
**Expected**	TACTG TGGTAAATCT::::::TGAAGACTAC CAGTGTACTAATTAT:::GGGCGCAGCCATGAG GCCAGGCACA::::::GCACATGCCT GTGGT	
Cl1-34	TACTG TGGTAAATCT::::::TGAAGACTAC CAGTGTACTAATTAT:::GGGCGCAGCCATGAG GCCAGGCACA::::::GCACATGCCT GTGGT	**HDR**
Cl35-36	TACTG TGGTAAATCT::::::TGAAGACTAC CAGTGTACTAATTAT::::::--:::::::::-------------- ---------------:::::::::::::::GCACATGCCT GTGGT	**3-NHEJ: 479bp del**
Clone37	TACTG TGGTAAATCT::::::TGAAGACTAC t-gcc--tggtaaatct::::::tgaagactac CAGTGTACTAATTAT:::::::::: :::::: ::::::::::::GGGCGCAGCCATGAG GCCAGGCACA::::::GCACATGCCT GTGGT	**5-NHEJ: 326bp ins**
Clone38	TACTG TGGTAAATCT::::::TGAAGACTAC ----------------------:::::--:::::::::::::::::::::::::::GGGCGCAGCCATGAG GCCAGGCACA::::::GCACATGCCT GTGGT	**5-NHEJ: 1191bp del**
Clone39	TACTG TGGTAAATCT::::::TGAAGACTAC tagccct agggctagtagtcttccaggtg::atttcacacaggaagac -------AGGCACA::::::GCACATGCCT GTGGT	**5,3-NHEJ: Backbone forward insertion**

Labels: Left homology arm; Replaced sequence in left arm; 2A-GFP-Wpre-ployA; Right homology arm

pD-GFP-sg-HA300-300bp (*PRDM14*)

	Left homology arm / 2A-GFP-Wpre-ployA / Right homology arm	
**Wild type**	CCATA AGGATGAGCA::::::TGAAGACTAC tagccct-- GCCAGGCACA::::::GAGTAGCCCT CAAGA	
**Expected**	CCATA AGGATGAGCA::::::TGAAGACTAC CAGTGTACTAATTATG::GGGCGCAGCCATGAG GCCAGGCACA::::::GAGTAGCCCT CAAGA	
Cl1-45	CCATA AGGATGAGCA::::::TGAAGACTAC CAGTGTACTAATTATG::GGGCGCAGCCATGAG GCCAGGCACA::::::GAGTAGCCCT CAAGA	**HDR**

Labels: Left homology arm; Replaced sequence in left arm; 2A-GFP-Wpre-ployA; Right homology arm

Supplementary Figure 6d: Knockin pattern analysis at the *PRDM14* locus. The left homology arm is indicated in yellow shadow and right HA in blue shadow. The inserted sequence is in red lowercase. Replaced sequence in left arm is indicated in purple. Sequences in grey indicate genomic DNA outside of homology arms.

WT	CTCCCACATGAAGTTTCATGAAGACTACTAGCCCT↓GCCAGGCACAATGACTCACGCCTGT	0
2#	CTCCCACATGAAGTTTCATGAAGACTACTAGCCCTtGCCAGGCACAATGACTCACGCCTGT	+1
3#	CTCCCACATGAAGTTTCATGAAGACTACTAGCCCTtGCCAGGCACAATGACTCACGCCTGT	+1
6#	CTCCCACATGAAGTTTCATGAAGACTAC-----------CTGCCAGGCACAATGACTCACGCCTGT	-5
9#	CTCCCACATGAAGTTTCATGAAGACTACTAGCC-----------AGGCACAATGACTCACGCCTGT	-5
10#	CTCCCACATGAAGTTTCATGAAGACTACTAGCC-T↓GCCAGGCACAATGACTCACGCCTGT	-1
12#	CTCCCACATGAAGTTTCATGAAG----------------------T↓GCCAGGCACAATGACTCACGCCTGT	-11

Supplementary Figure 7: Presence of indels at the non-target allele of all iPSC clones with heterozygous HDR knockin at the *PRDM14* locus. The non-targeted allele was amplified by PCR, followed by Sanger sequencing to identify indels.

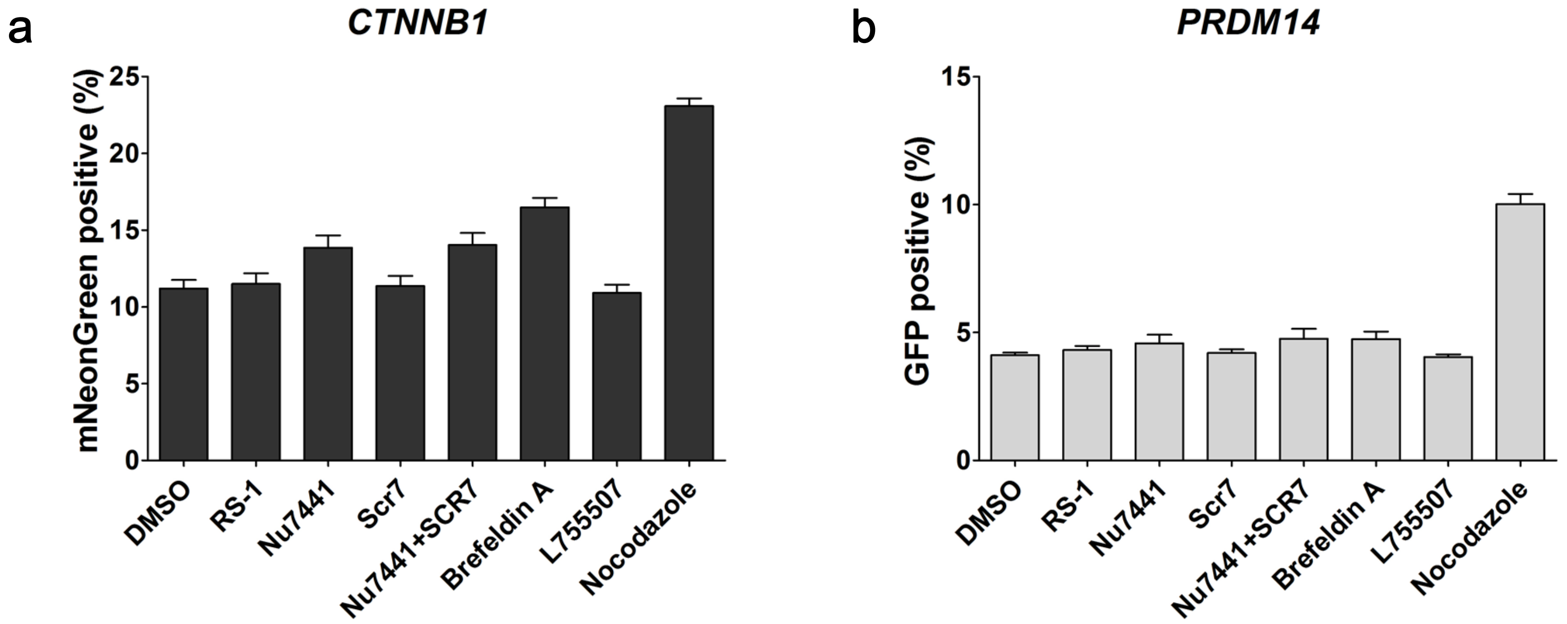

Supplementary Figure 8: The effects of small molecules on HDR in the H1 ES cell line. (**a-c**) The effects of small molecules on HDR efficiency at the CTNNB1 locus (**a**) or PRDM14 locus (**b**) . The human H1 ES cells were treated with RS-1 (10μM), Nu7441 (2μM), SCR7 (1μM), Brefeldin A (0.1μM), L755507 (5μM) or Nocodazole (100ng/ml) at 0-24h after nucleofection and the HDR efficiency was determined by FACS on Day 3.

Supplementary Fig.9a

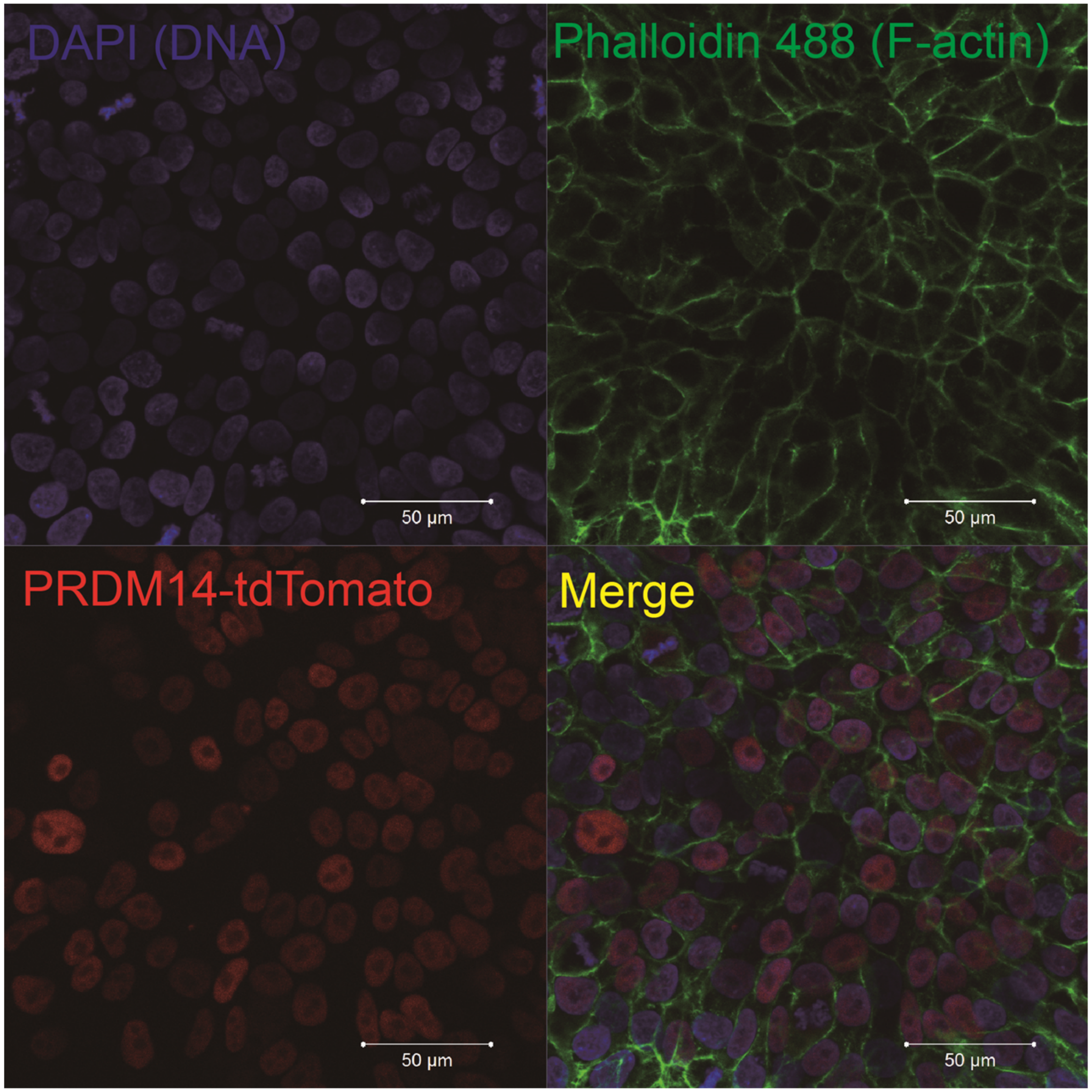

Supplementary Fig.9b

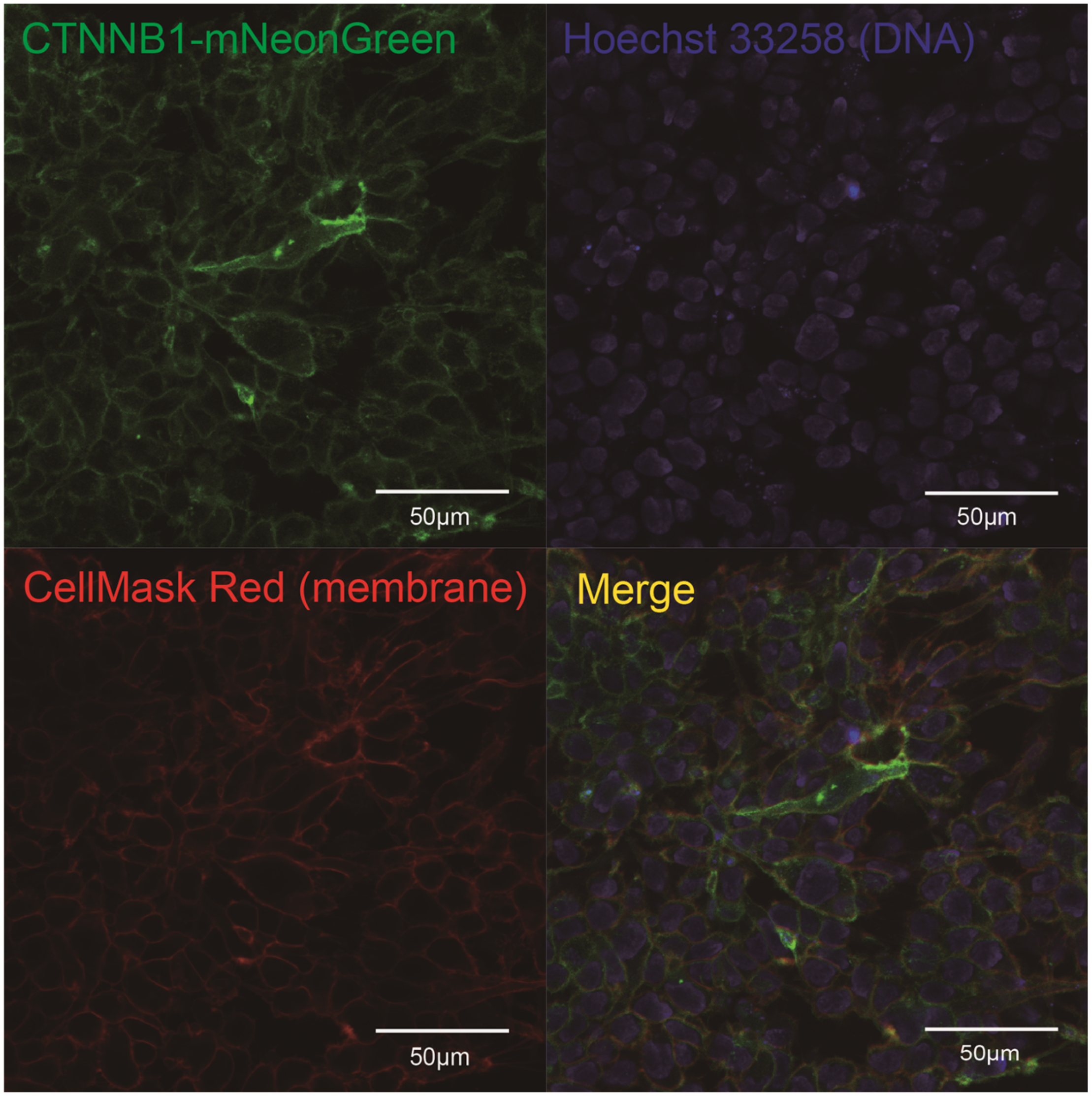

Supplementary Figure 9: Illustration of cellular location of genes of interest by fusion with a fluorescent protein after HDR targeting in iPSCs.

(**a**) Localizaiton of PRDM14 at the nucleus as indicated by a tdTomato reporter. A new donor plasmid was created to generate a fusion gene of PRDM14 and tdTomato, which are separated by a GS linker The cells were stained with DAPI and Phalloidin 488 to show the nucleus and plasma membrane. (**b**) Localizaiton of CTNNB1 at plasma membrane as indicated by a mNeonGreen reporter. The cells were stained with Hoechst 33258 and CellMask Red to show the nucleus and menbrane. (scale bar = 50 μm).

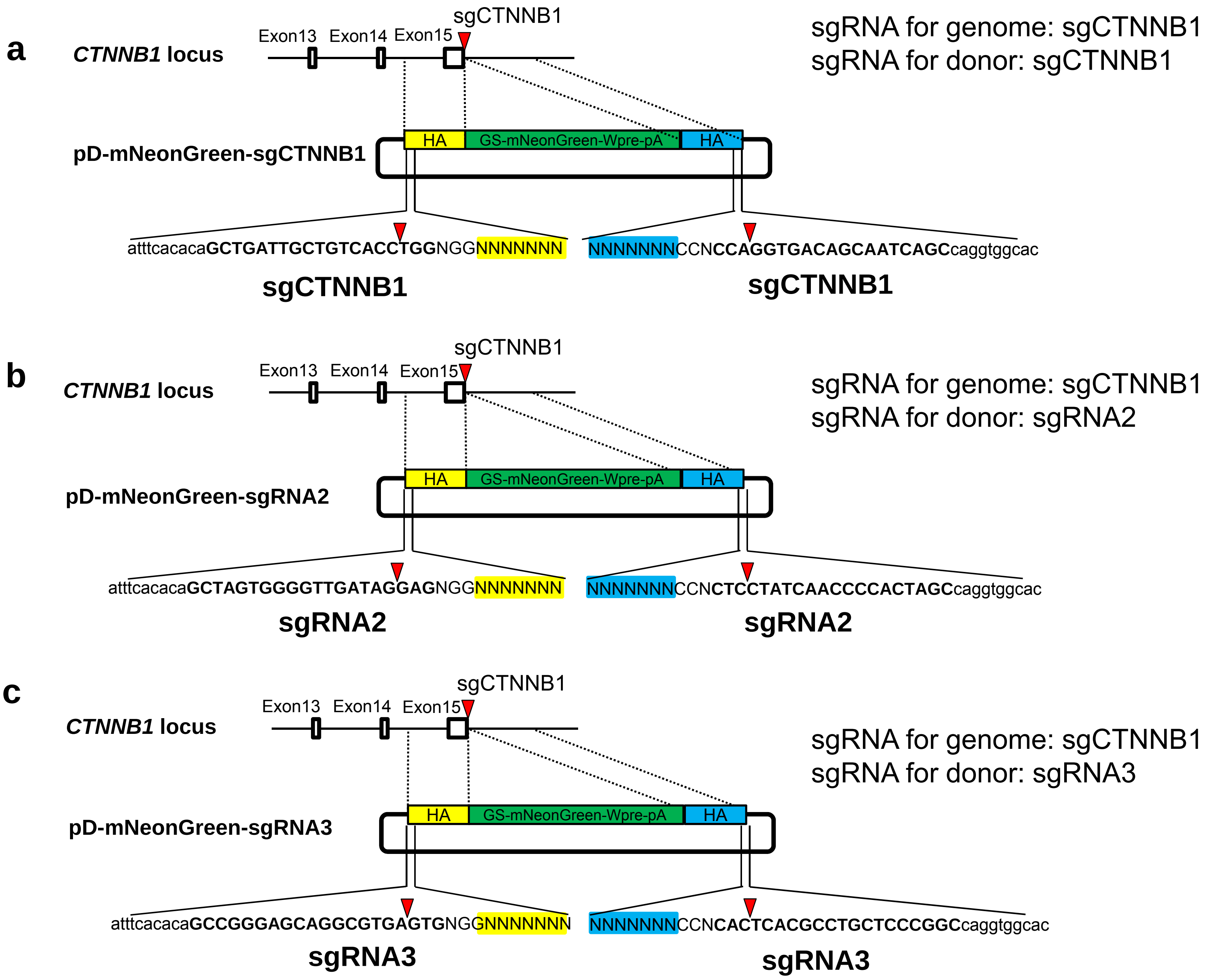

a
CTNNB1 locus
Exon13
Exon14
Exon15
sgCTNNB1
pD-mNeonGreen-sgCTNNB1
HA
GS-mNeonGreen-Wpre-pA
HA
atttcacacaGCTGATTGCTGTCACCTGGNGGNNNNNNN
NNNNNNNCCNCCAGGTGACAGCAATCAGCcaggtggcac
sgCTNNB1
sgCTNNB1
sgRNA for genome: sgCTNNB1
sgRNA for donor: sgCTNNB1
b
CTNNB1 locus
Exon13
Exon14
Exon15
sgCTNNB1
pD-mNeonGreen-sgRNA2
HA
GS-mNeonGreen-Wpre-pA
HA
atttcacacaGCTAGTGGGGTTGATAGGAGNGGNNNNNNN
NNNNNNNCCNCTCCTATCAACCCCACTAGCcaggtggcac
sgRNA2
sgRNA2
sgRNA for genome: sgCTNNB1
sgRNA for donor: sgRNA2
c
CTNNB1 locus
Exon13
Exon14
Exon15
sgCTNNB1
pD-mNeonGreen-sgRNA3
HA
GS-mNeonGreen-Wpre-pA
HA
atttcacacaGCCGGGAGCAGGCGTGAGTGNGGNNNNNNN
NNNNNNNCCNCACTCACGCCTGCTCCCGGCcaggtggcac
sgRNA3
sgRNA3
sgRNA for genome: sgCTNNB1
sgRNA for donor: sgRNA3

d

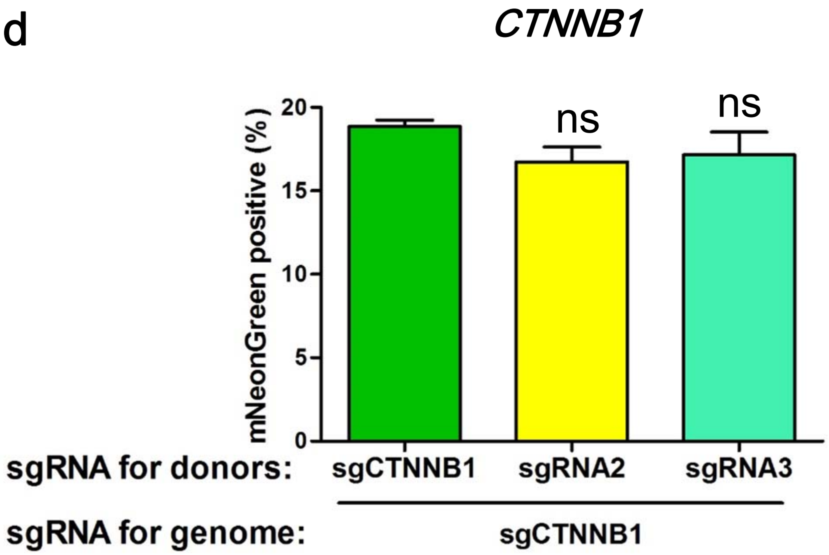

Supplementary Figure 10: The effects of using one vs. two sgRNAs in the double cut donor system on HDR efficiency. (**a-c**) Schematic outline of genome editing at the *CTNNB1* locus using double cut HDR donors, which will be linearized by Cas9 and sgCTNNB1 (**a**), sgRNA2 (**b**), or sgRNA3 (**c**), respectively. The donors contain a GS-mNeonGreen-Wpre-polyA sequence sandwiched by left homology arm (HA, marked in yellow shadow) and right homology arm (HA, marked in blue shadow). (**d**) The HDR efficiency was determined by FACS at day3 after co-transfection with Cas9/sgRNA and one of the three pD-mNeonGreen-sg donors (**a-c**). Statistical significance was calculated using the Student's paired t-test: ns, not significant.
